# Going Deep into the Surface Chemistry of Carbon Dots: Influence of Functional Groups on the Redox Abilities

**DOI:** 10.1002/smll.202514420

**Published:** 2026-03-03

**Authors:** Laura Morbiato, Maria Sbacchi, Jacopo Dosso, Paolo Pengo, Pierangelo Gobbo, Giacomo Filippini, Maurizio Prato

**Affiliations:** ^1^ Department of Chemical and Pharmaceutical Sciences INSTM UdR Trieste University of Trieste Trieste Italy; ^2^ Center for Cooperative Research in Biomaterials (CIC biomaGUNE) Basque Research and Technology Alliance (BRTA) Donostia‐San Sebastián Spain; ^3^ Ikerbasque Basque Foundation for Science Bilbao Spain

**Keywords:** carbon dots molecular weight, carbon dots (photo)redox properties, carbon dots supramolecular interactions, nanomaterials chemistry, purification and characterization

## Abstract

Carbon dots (CDs) are emerging nanomaterials with low‐cost synthesis and highly tunable surface chemistry, which have propelled their widespread use in catalysis and photocatalysis. Yet, unraveling their chemical structure remains essential to unlocking their full potential. Here, we systematically investigated the redox behavior of three amine‐rich CD batches synthesized from either pure *L*‐arginine or mixtures of *L*‐arginine with alkyl diamines (ethylenediamine or putrescine), yielding different surface amine densities. Strikingly, the density and accessibility of surface amines were found to directly govern CD (photo)redox activity, evaluated through the reduction of resazurin to resorufin. CDs enriched in surface amines exhibited markedly superior performance under both dark and illuminated conditions. Electrochemical studies confirmed electron transfer feasibility in both scenarios. Control experiments with molecular amines showed no activity, highlighting the indispensable role of supramolecular CD‐substrate interactions, likely involving at least two vicinal amines, in enabling electron transfer. The presence of these interactions was confirmed by both ^1^H‐NMR spectroscopy and isothermal titration calorimetry. Furthermore, absolute CD molecular weights determined by multi‐detection gel permeation chromatography enabled precise stoichiometric analysis. Together, these findings establish a framework for tailoring CD surface chemistry to achieve controlled electron transfer, opening new opportunities across (nano)materials science, organic synthesis, and chemical biology.

## Graphical abstract

The chemical structure of Carbon Dots (**CDs**) is still a mystery despite years of study. Herein, we show how **CDs** with a higher density of surface‐accessible amines display enhanced (photo)redox performance. Electrochemical and thermodynamic analyses confirmed the feasibility of electron transfer, while mechanistic investigations revealed that the supramolecular binding between **CDs** and resazurin acts as the gateway to efficient electron shuttling.

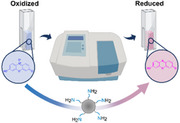

## 1 Introduction

Over the past two decades, carbon dots (CDs) have attracted significant attention owing to their low‐cost, straightforward synthesis and wide‐ranging potential applications [1]. To fully harness their versatility, however, a deeper understanding of their chemical structure is essential [2, 3]. Structurally, CDs are often described as core‐shell‐like nanoparticles (typically <10 nm) comprising a rigid inner core surrounded by a corona of diverse functional groups, including amines, alcohols, phenols, and carboxylic acids [4, 5]. CDs can be produced by either top‐down or bottom‐up methods, with the latter emerging as the preferred route, due to their ability to preserve the functionalities of the molecular precursors [6, 7]. In this way, the intrinsic chemical properties of the starting materials can be directly translated into the final nanostructure [8, 9, 10, 11, 12]. For example, nitrogen‐rich precursors such as amino acids and amines yield amine‐rich CDs [13].

Surface amines play a pivotal role in dictating the chemical behavior of **CDs**. They mediate interactions with the environment, impart water solubility, and, importantly, modulate redox activity. Although **CDs** exhibit complex surface chemistry, previous characterization indicated that carboxylic acids, hydroxyl groups, imines, and heterocyclic nitrogen‐containing functional groups are present on their surfaces. However, their low concentration suggests that they play a minor role compared to amino groups in determining the overall reactivity of **CDs** [14]. For this reason, amine‐rich **CDs** have thus emerged as effective, metal‐free (photo)catalysts for valuable organic transformations under mild conditions [13, 15, 16]. In some cases, surface amines promote noncovalent interactions, such as hydrogen or halogen bonding, between **CDs** and organic substrates, giving rise to nanometric supramolecular complexes capable of driving otherwise inaccessible transformations [17, 18, 19, 20, 21]. These binding events can be quantitatively characterized by isothermal titration calorimetry (ITC) and nuclear magnetic resonance (NMR) spectroscopy, which provide binding constants and thermodynamic parameters (*ΔH*, *ΔG*, *ΔS*) [18, 22, 23, 24]. Accurate interpretation of these data requires precise **CD** molar concentrations, which depend on molecular weight. Multi‐detection gel permeation chromatography (MD‐GPC) offers a powerful solution, enabling absolute molecular weight determination without calibration curves while simultaneously revealing polydispersity and hydrodynamic dimensions [25]. From these measurements, the number of surface amines per **CD** can be calculated, providing crucial molecular‐level insights into **CD** reactivity.

In this study, we performed a comprehensive structural and functional analysis of three batches of amine‐rich **CDs** prepared from *L*‐arginine alone or from mixtures of *L*‐arginine with an alkyl diamine (ethylenediamine or putrescine) [26, 27]. As a redox benchmark, we focused on the reduction of resazurin (7‐hydroxy‐3*H*‐phenoxazin‐3‐one‐10‐oxide) to resorufin (7‐hydroxy‐3*H*‐phenoxazin‐3‐one), a transformation typically catalyzed by cellular enzymes but also achievable by small‐molecule reductants or photoredox catalysts [28, 29, 30, 31]. Because of its simplicity and reliability, the resazurin assay provides a robust readout of redox activity across diverse systems, from living cells to nanoparticles [32, 33, 34]. In addition, despite being resazurin a well‐known redox agent, to the best of our knowledge, this work is one of the few examples where this molecule is used to probe the reactivity of **CD** surface, helping to gain insights into the elusive surface chemistry of these complex nanomaterials.

**FIGURE 1:**
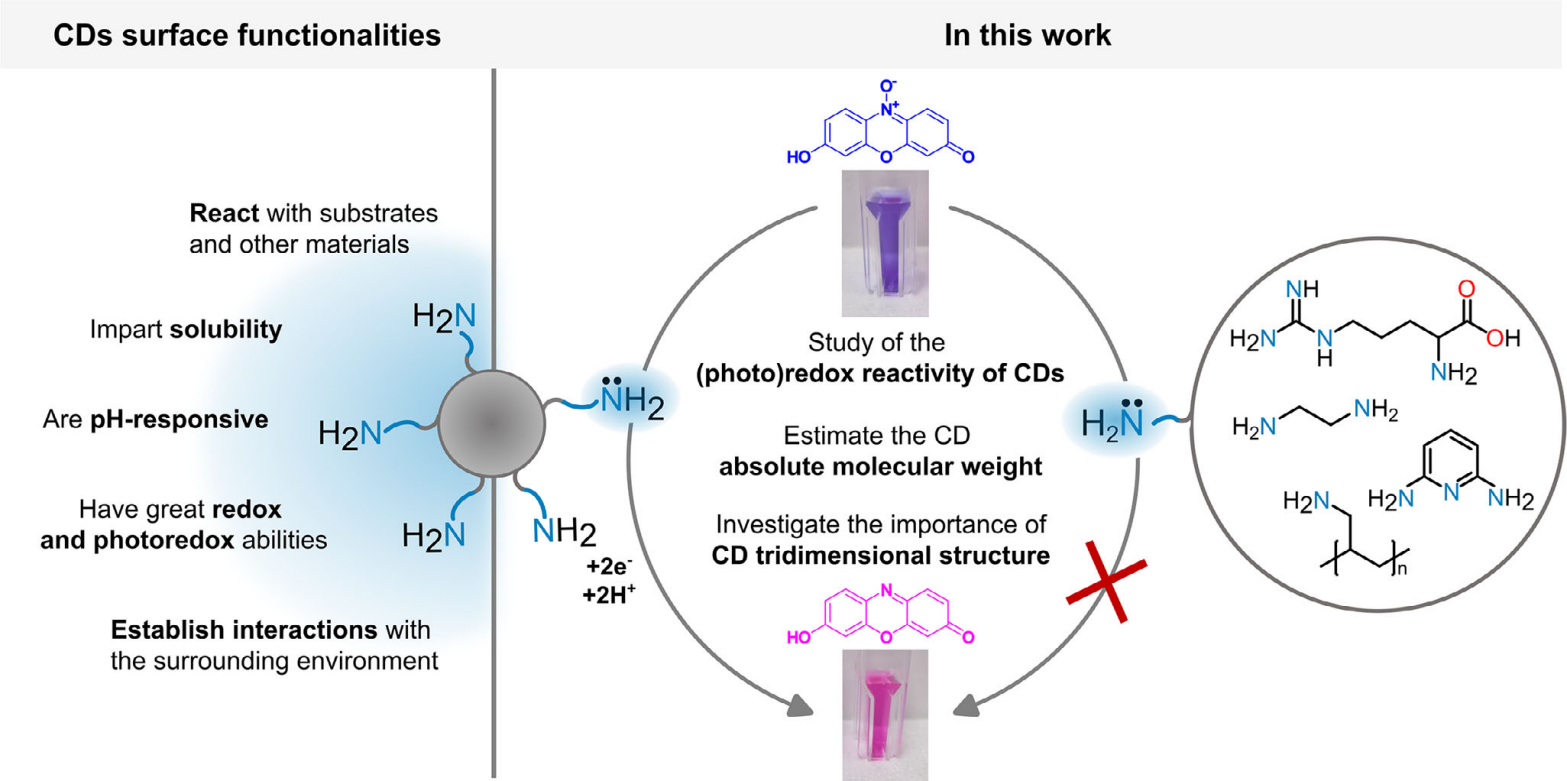
Schematic representation of the **CD‐**mediated reduction of resazurin (purple blue, top) to resorufin (pink, bottom) studied in this work. The photographs show cuvettes containing aqueous solutions of resazurin or resorufin in carbonate buffer (0.1 M, pH 9.4, conc. = 2 × 10^−6^ M). The main properties of the surface functionalities of **CDs** are highlighted on the left. The amine‐bearing model molecules tested, which are ineffective as reducing agents in the resazurin reduction are reported on the right.

Our results reveal that the precursor choice strongly influences **CD** surface chemistry and, consequently, redox performance. **CDs** synthesized from the combined use of starting *L*‐arginine and putrescine (the longest diamine employed) exhibited the highest density and accessibility of surface amines, correlating with superior redox efficiency under both dark and illuminated conditions. Electrochemical analyses confirmed the thermodynamic feasibility of electron transfer, with negative Gibbs free energy values for both thermal (*ΔG_ET_*) and photoinduced (*ΔG_PET_*) pathways [35]. Control experiments with small amine model molecules showed no activity, underscoring the importance of supramolecular **CD‐**substrate interactions. ITC and NMR binding studies further revealed that such interactions, likely involving at least two vicinal amines, are necessary to promote electron transfer, whereas isolated functional groups, such as the molecular controls, are insufficient (Figure 1).

Overall, this work demonstrates how **CD** surface chemistry dictates redox behavior and highlights the critical role of supramolecular interactions in enabling electron transfer. By combining MD‐GPC molecular weight determination with thermodynamic binding analyses, we establish a framework for quantitatively linking **CD** surface functionality to reactivity. These findings provide fresh insights into the elusive chemical nature of **CDs** and open new avenues for their rational nanoengineering for applications ranging from optoelectronics to catalysis, materials science, and chemical biology [10, 36, 37].

## 2 Results and Discussion

### 2.1 Synthesis and Characterization of CDs

**FIGURE 2:**
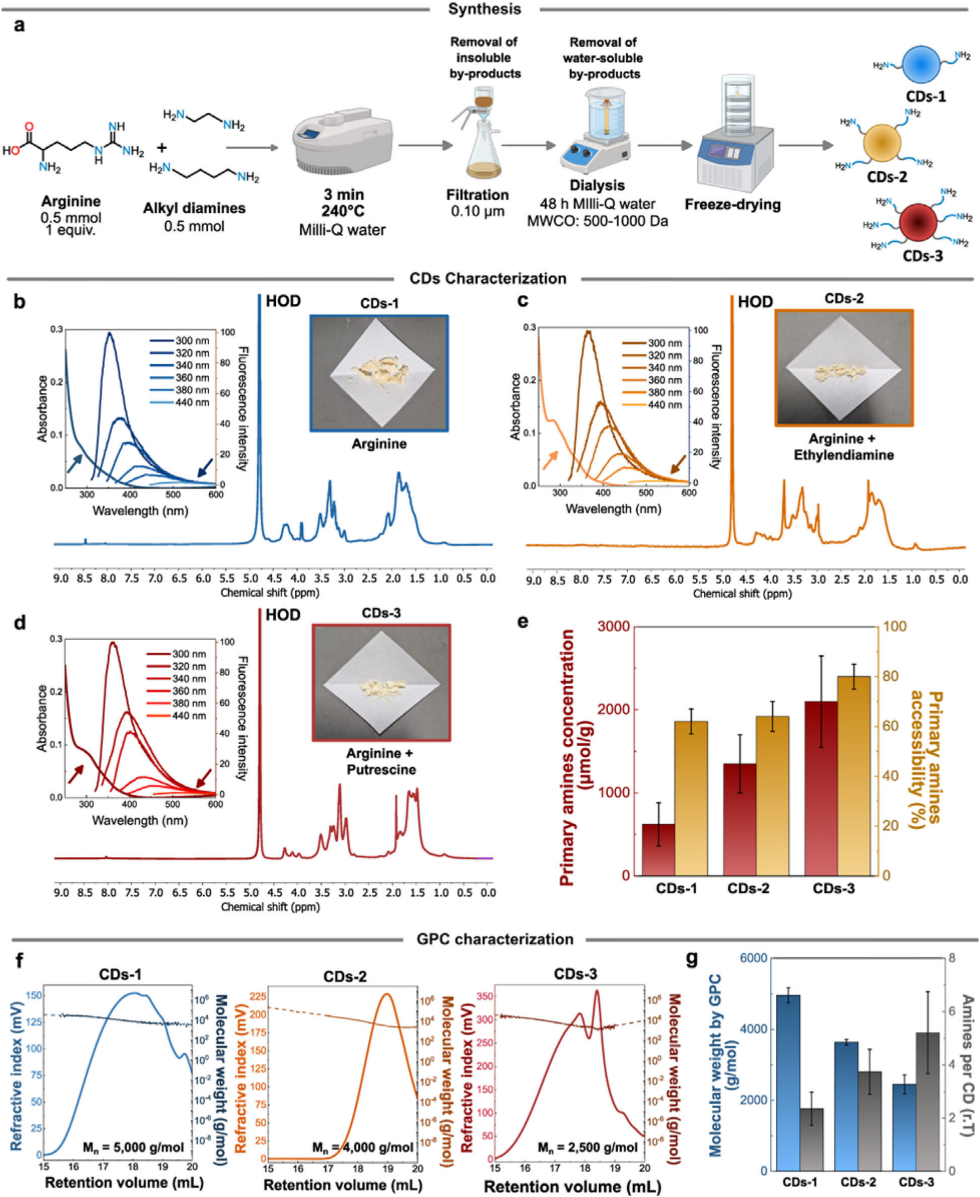
a) Scheme showing the synthesis and purification of **CDs 1‐3** starting from either pure *L*‐arginine or mixtures of *L*‐arginine and an alkyl diamine (ethylenediamine or putrescine). b‐d) Characterization of **CDs 1‐3** including ^1^H‐NMR spectrum, absorbance spectrum and emission spectra (excitation wavelengths are shown in the figure). The insets pictures show the typical aspect of purified and lyophilized **CDs**. All the ^1^H‐NMR spectra were acquired in D_2_O and calibrated against residual water. Absorbance and emission spectra were acquired in Milli‐Q water at the concentration of 10^−1^ mg/mL. **e)** Primary amine content (µmol/g) determined by Kaiser test and corresponding accessibility percentages for **CDs 1‐3**. Error bars refer to the standard deviation over three independent tests of the same **CDs** batch. **f)** MD‐GPC chromatograms for **CDs 1‐3** showing the refractive index signal and the calculated molecular weight trend (the dashed part of the plot indicates predicted values). g) Plot comparing the number average molecular weight (*M_n_*) and the number of accessible surface amines at room temperature for **CDs 1‐3**. Error bars refer to the standard deviation over three independent GPC injections of the same **CDs** batch.

The **CDs** object of this work, namely CDs 1‐3, were synthesized *via* microwave assisted bottom‐up hydrothermal synthesis following a previously established procedure (Figure 2a, Experimental Section Synthesis of **CDs**) [38]. Specifically, CDs‐1 were obtained from pure *L*‐arginine, CDs‐2 were obtained from an equimolar mixture of *L*‐arginine and ethylenediamine, while CDs‐3 were obtained from an equimolar mixture of *L*‐arginine and putrescine.

After their synthesis, **CDs 1‐3** underwent filtration followed by dialysis purification against Milli‐Q water, and were then characterized by ^1^H‐NMR spectroscopy, UV–vis spectroscopy, and fluorescence spectroscopy (Figure 2b–d). Post‐dialysis ^1^H‐NMR spectra displayed broad signals consistent with the polymeric nature of **CDs** and no sharp signals, confirming the absence of residual small molecules derived from the synthesis. This confirmed successful synthesis and purification of **CDs** [39].

UV–vis absorption and emission measurements indicate that the three **CD** samples had comparable optoelectronic properties, with the typical excitation‐dependent emission profiles characteristic of this class of nanomaterials (Figure 2b–d, Experimental Section Characterization of **CDs**) [40]. In particular, **CDs 1‐3** display a strong absorption in the UV range, with a tail in the visible region of the spectrum (up to 450 nm) and a maximum of emission centered at 350 nm (*λ_exc_* = 300 nm).

After this initial characterization, the surface chemistry of **CDs** was investigated, focusing specifically on primary amines. These functional groups were quantified using the Kaiser test (KT), a colorimetric assay that detects aliphatic primary amines through formation of Ruhemann's purple dye upon reaction with ninhydrin at 120°C [41]. To assess amine accessibility, KT was performed at room temperature, where only accessible and sterically unhindered amines react with ninhydrin [14]. Comparison with results obtained at 120°C enabled estimation of the percentage of accessible surface amines relative to the total population (Figure 2e) [42].

Interestingly, both the quantity and accessibility of surface amines were found to depend on the presence and structure of the alkyl diamine precursor [14, 38]. Specifically, **CDs‐3** (synthesized from *L*‐arginine and putrescine) exhibited the highest number of accessible primary amines (1680 ± 310 µmol/g), followed by **CDs‐2** (*L*‐arginine and ethylenediamine – 864 ± 160 µmol/g) and **CDs‐1** (*L*‐arginine only – 384 ± 120 µmol/g) (Figure 2e).

After the initial characterization, a MD‐GPC system was used to analyze the **CDs** and estimate their absolute molecular weights, molecular weight distributions, and quantify their purity (Figure 2f,g; Figures S1–S3, Table S1, Experimental Section Multi‐detection gel permeation chromatography (MD‐GPC)). Among the experimental parameters reported in Table S1, in this study, *M_n_* was taken as the reference molecular weight of the **CDs**. Notably, all the measured *M_n_* values were within the same order of magnitude and showed a decreasing trend: 5000 g/mol for **CDs‐1**, 4000 g/mol for **CDs‐2**, and 2500 g/mol for **CDs‐3**. In addition, GPC analyses revealed narrow size distributions for all **CDs**, with polydispersity indices *(Ð)* close to 1, indicating uniform nanoparticle dimensions with minimal size variation [43].

Importantly, the choice of starting materials influenced both surface amine properties and the overall chemical structure of the **CDs**. While previous AFM analyses indicated that **CDs 1‐3** are characterized by quasi‐spherical morphologies with diameters ranging from 2 to 4 nm for all samples [38], hydrodynamic radii (*R_H_*) determined by MD‐GPC revealed that **CDs‐1** were slightly larger (1.7 ± 0.1 nm) than **CDs‐2** and **CDs‐3** (both 1.4 ± 0.1 nm) (Table S1). In addition, dynamic light scattering (DLS) measurements confirmed the decreasing dimensions of **CDs 1‐3** (Figure S4, Experimental Section Dynamic light scattering), in accordance with the GPC results. MD‐GPC analyses showed that larger **CDs** also exhibited higher molecular weights, with **CDs‐1** reaching 5000 g/mol.

The absolute number of surface amines per nanoparticle could then be calculated by combining molecular weight data with KT‐determined amine concentrations. This analysis revealed a progressive increase from **CDs‐1** to **CDs‐3** (Figure 2g), demonstrating that **CDs‐3** not only exhibited the highest amine content per gram of material (2100 ± 550 µmol/g, based on KT at 120°C) but also the greatest number of surface amines per individual nanoparticle (5 ± 1), compared to **CDs‐1** (2 ± 1) and **CDs‐2** (3 ± 1).

### 2.2 Evaluation of CDs Redox Abilities

**FIGURE 3:**
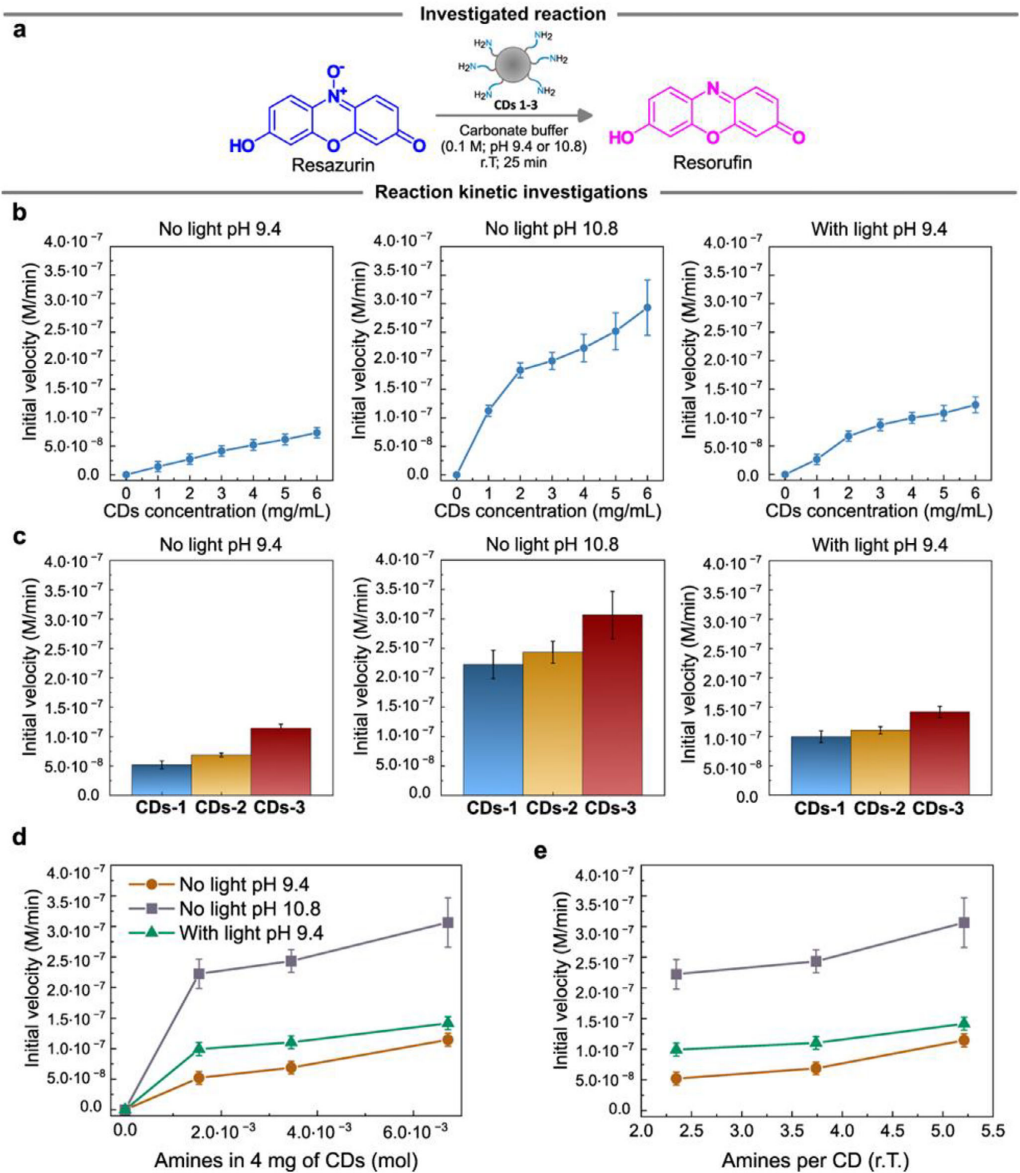
**a)** Reaction scheme for resazurin reduction by **CDs**. **b)** Initial velocities for **CDs‐1** mediated resazurin reduction in carbonate buffer (0.1 M, pH 9.4 or 10.8) with and without light irradiation (Kessil lamp, *λ_max_* = 427 nm, 45 W, Irradiance = 200 mW/cm^2^). Reactions were monitored by UV–vis spectrophotometry in a polymethylmethacrylate (PMMA) semi‐micro cuvettes (1 mL volume) with constant resazurin concentration (2 × 10^−6^ m) and variable **CDs** concentration as indicated. **c)** Initial velocities for **CDs 1‐3** mediated resazurin reduction under identical conditions (**CDs** concentration 4 mg/mL, resazurin concentration 2 × 10^−6^ M). d) Plot showing the initial velocities reported in **(c)** versus the total moles of amines in the reaction mixture for each batch of **CDs** (the amounts of amines were calculated from the KT values at room temperature). **e)** Plot showing the initial velocities reported in **(c)** versus the number of amines on the surface of each **CD**. These numbers were calculated considering the molecular weight of **CDs** retrieved by GPC and the KT values at room temperature. All the initial velocities of the reactions were retried by plotting the resorufin concentration versus time, and by fitting the first linear part of the kinetic as described in the Experimental Section Reduction of resazurin. Error bars refer to the standard deviation over three replicas.

The reduction of resazurin to resorufin served as a model reaction to assess the redox properties of the three **CD** batches (Figure 3a; Figures S5–S7, Experimental Section Reduction of resazurin). This reduction was monitored by UV‐Visible spectroscopy, tracking the resazurin color change from blue (*λ_max_* = 600 nm) in the oxidized form, to pink (*λ_max_* = 570 nm) in the reduced form [44], with molar extinction coefficients reported in Table S2. Since resazurin exhibits pH‐dependent optical properties in addition to redox‐state variations, it is necessary to work in a pH‐controlled environment [45]. To maintain pH control, and according to the chemical nature of the functional groups on the surface of the **CDs**, all reactions were performed in carbonate buffer under alkaline conditions. In fact, previous reports showed that **CDs 1‐3** in Milli‐Q water have a native pH of 9.0, 9.2, and 9.5, respectively [38]. For this reason, pH 9.4 was chosen as the reference pH for both redox and photoredox kinetic assessments.

To determine the minimal loading of **CDs‐1** required for efficient resazurin reduction, the (photo)redox abilities of **CDs‐1** were assessed at a concentration of 10, 50, 75, and 100 mol% with respect to resazurin concentration (2 × 10^−6^ M). The assessments were conducted both in the dark and under light irradiation. For the photoredox experiments a Kessil lamp was used as the visible light source (*λ_max_* = 427 nm, 45 W, Irradiance = 200 mW/cm^2^) at its maximum power, and in the absence of band filters. In the dark, the reaction kinetics are very slow, and resazurin reduction can take days to complete. For this reason, the reactions in the dark were monitored for 4 days. On the other hand, reactions in the light were found to be faster, and were monitored for 24 h (Figure S8). Unfortunately, during these experiments with prolonged irradiation time, stability assessments of the resazurin and resorufin showed that both these dyes undergo photodegradation under the operational conditions. In addition, time‐dependent UV–vis spectra showed a progressive decrease in resorufin absorbance, confirming that, under light irradiation, the resorufin photodegradation rate exceeds the resazurin reduction rate. Based on these experimental observations, to shorten the reaction times and minimize resorufin degradation, the evaluation of the (photo)redox abilities of **CDs 1‐3** was conducted with a large excess of **CDs** compared to resazurin.

Figure 3b reports the initial velocities (M/min) of the resorufin production for the tested **CDs** in the dark. Reagent stability in the selected buffers was confirmed prior to kinetic measurements (Figure S9).

The redox assessments were conducted in carbonate buffer 0.1 M at pH 9.4 with a 2 × 10^−6^ M resazurin concentration, and gradually increasing **CDs‐1** concentration from 1 to 6 mg/mL. This resulted in an increase of the initial velocities of the reactions, ranging from 1.42 × 10^−8^ to 7.36 × 10^−8^ M/min (Figure S10). Subsequently, to ensure complete amine deprotonation, and to evaluate the influence of these functional groups on the reaction kinetic, the pH was increased to 10.8.

Notably, increasing the pH from 9.4 to 10.8 and repeating the experiments with the same concentrations of **CDs‐1**, resulted in faster kinetics and showed the same increasing trend depending on the **CD** concentration (Figure S11). Notably, previous studies have reported that the highest buffering capacity of **CDs 1‐3** ranges between pH 6 and 8 [14]. This indicated the presence of multiple functional groups with pKa values within that range. However, these values are inconsistent with those of simple aliphatic amines, which typically have pKa values between 9 and 11 [38]. This inconsistency can be explained by the spatial proximity of non‐protonated amines close to vicinal protonated groups. In fact, as observed for alkyl diamines, the protonation of the first amine group significantly affects the pKa of the second one, causing it to decrease. For instance, the first pKa of ethylenediamine is 10.7, while the second drops to 6.9 [14]. The enhanced reactivity of **CDs** at higher pH, when amines are deprotonated and bear a lone electron pair, further supports the central role of these functional groups in the redox activity of **CDs**.

Following these initial redox assessments, the performance of **CDs‐2** and **CDs‐3** was compared to that of **CDs‐1**, at the chosen **CD** concentration of 4 mg/mL, allowing for the evaluation of the impact of the differential surface amine density of **CDs 1‐3**. Under these experimental conditions, **CDs 1‐3** showed a progressive increase in their abilities to reduce resazurin, reaching an initial velocity up to 3.06 × 10^−7^ M/min for **CDs‐3** at pH 10.8 (Figure 3c).

Subsequently, the photoredox abilities of **CDs 1‐3** were evaluated. The photoredox assessments were carried out at the chosen reference pH of 9.4, which is around the native pH of the nanoparticles, by repeating the reactions under light irradiation (Figure S12). A Kessil lamp was used as the visible light source (*λ_max_* = 427 nm, 45 W, Irradiance = 200 mW/cm^2^) at its maximum power, and in the absence of band filters. Prior to performing the kinetic assessments, the stability of resazurin, resorufin, and **CDs** under light irradiation was examined, confirming the overall good stability of all the reagents in these operative conditions (Figure S9). Notably, the same increasing trend depending on the **CDs‐1** concentration was observed (Figure 3b). The photoredox abilities of **CDs‐1** were then compared to those of **CDs‐2** and **CDs‐3** with a 4 mg/mL concentration of **CDs** at pH 9.4 (Figure 3c).

When light was shed on **CDs 1‐3**, higher initial velocities were observed, which is consistent with **CDs** in their excited state (CDs*) being better redox agents than **CDs** in their ground state [46, 47]. Additionally, we estimated the quantum yield (QY) of the photochemical reaction (Table S3 and Figure S13) to better understand the photochemical processes involved in the reduction of resazurin. As described in Experimental Section Quantum yield (QY) estimation, the quantum yield of the reaction was estimated to be ≈1 × 10^−8^. It is worth mentioning that for a photochemical process in which the absorption of a single photon results in the formation of one molecule of product, the theoretical maximum QY is equal to 1 [48]. Since the QY for the reduction of resazurin is much smaller than 1, it is reasonable to assume that no radical chain mechanisms are involved [49].

Figure 3d shows the initial velocities of a given reaction in relation to the exact number of accessible amines in solution. This number was calculated by considering the number of amines available at room temperature, as determined by KT (**CDs** concentration = 4 mg/mL). The comparison revealed that an increase in the number of available amines in the solution resulted in an increase in the initial velocities of the resazurin reduction, which is consistent with the enhanced redox activity observed for **CDs 1‐3**. Additionally, the increase in the initial velocities could be correlated with the increase in amines present on the surface of a single **CD** (Figure 3e). These results demonstrated that identical amounts of **CDs** may possess different numbers of redox active sites, underscoring the importance of thoroughly characterizing the **CDs** before drawing conclusions about their redox performance.

Because of the structural complexity of **CDs**, it is not possible to unambiguously determine the exact number of redox equivalents provided by each individual particle. Similarly, identification of specific chemical bonds formed on the **CD** surface after electron transfer is challenging. However, since we ascribed the redox activity of the **CDs** to their surface amine groups, a fair assumption is that, after electron transfer, these surface amines may suffer of oxidative degradation [50], probably leading to a partial degradation of the functional groups on the **CD** surface. In order to gain more insights into this aspect and study the chemical changes that may happen on the surface of the **CDs** after the resazurin reduction, **CDs‐1** were recovered after the redox assessments, and their UV–vis spectrum was compared to that of the pristine **CDs‐1**. As reported in Figure S14, the two absorption spectra were comparable, and only minor changes were observed. This was ascribed to the large stoichiometric excess of **CDs‐1** (≈10^−3^ m) with respect to resazurin (≈10^−6^ M), which made it difficult to observe a degradation of the **CDs** involved in the electron transfer.

To complete the characterization of **CDs 1‐3**, their Zeta‐potential in the reaction environment (carbonate buffer 0.005 M, pH 9.4) was measured (See Experimental Section Zeta‐potential measurements). As shown in Figure S15, all the **CDs** exhibited a negative Zeta‐potential at alkaline conditions (−6.9 ± 1.3 mV, −9.6 ± 1.2 mV, and −7.6 ± 1.4 mV for **CDs 1‐3**, respectively). These negative potentials in a basic aqueous solution suggested that the amines and the other pH‐sensitive groups, such as carboxylic acids and phenols on the surfaces of **CDs 1‐3** were deprotonated, making the **CDs** good reducing agents. Additionally, all **CDs** exhibited similar Zeta‐potentials within the experimental error, suggesting that the surface charge of **CDs** is not a key factor in dictating their (photo)redox performance.

To further confirm the results of the kinetic assessments, **CDs 1‐3** were also electrochemically characterized in *N,N*‐dimethylformamide (DMF) with 0.1 M of tetrabutylammonium hexafluorophosphate (TBAPF_6_). A saturated calomel electrode (SCE) was used as the reference electrode. See Figure S16 and Experimental Section Cyclic voltammetry for all the experimental details [51]. The results of the electrochemical measurements are summarized in Table 1.

**TABLE 1:** Table summarizing the results of electrochemical characterization of **CDs 1‐3** and *ΔG_ET_* and *ΔG_PET_* calculation. *E^ox^* (**CDs**/**CDs** ^+^) was retrieved by cyclic voltammetry; *E_00_* (**CDs**/CDs*) was spectroscopically estimated from the tail of the absorbance spectrum of **CDs**; E* (**CDs** ^+^/CDs*) was calculated according to Rehm–Veller equation (Equation 5, Experimental Section Cyclic voltammetry); *ΔG_ET_* was retrieved according to Equation 6 (Experimental Section Cyclic voltammetry); and *ΔG_PET_* was retrieved according to Equation 8 (Experimental Section Cyclic voltammetry). **CDs** ^+^ refers to the oxidized form of **CDs**. CDs* refers to the excited states of **CDs**. *E^RED^* (resazurin/resorufin) = −0.29 V *vs* SCE [52].

	*E^OX^* (CDs/CDs^+^) V	*E_00_* (CDs/CDs*) V	E* (CDs^+^/CDs*) V	*ΔG_ET_* kcal/mol	*ΔG_PET_* kcal/mol
**CDs‐1**	+1.27	2.72	−1.45	−22.60	−26.75
**CDs‐2**	+1.30	2.77	−1.47	−23.29	−27.21
**CDs‐3**	+1.34	2.82	−1.48	−24.21	−27.44

Given that the reduction potential of resazurin (−0.29 V *vs* SCE) is less negative than that of **CDs** (*ca*. −1.30 V *vs* SCE), the results reported in Table 1 confirmed the feasibility of electron transfer between **CDs** and resazurin [52]. In addition, the redox potentials **CDs 1‐3** were found to be comparable (Figure S16). This indicated that the surface amines of **CDs 1‐3** share a similar electrochemical nature, and that the observed differences in their reduction efficiency toward resazurin were solely due to variations in the number and accessibility of these surface groups, rather than to differences in their electrochemical properties. Notably, these reduction potentials were found to be similar to those of molecular amines such as phenethylamine and benzylamine (+1.20 V *vs* SCE), highlighting the strong analogy between the aminic groups exposed on the **CD** surface and these small molecular amines [53].

To gain further insight into the reaction mechanism, we performed some control experiments. Specifically, we tested the redox abilities of small molecules and polymers that mimic the surface functionalities of **CDs**. We carried out the resazurin‐to‐resorufin reduction reaction in the presence of small aliphatic amines (such as ethylenediamine, putrescine and butylamine), molecules bearing both aminic and acidic moieties (arginine and 12‐aminododecanoic acid), carboxylic acids (formic acid), aromatic amines (benzylamine and phenethylamine), heterocycles (pyridine, 2,6‐pyridinedicarboxylic acid, 2,6‐diaminopyridine) and small amine‐rich polymer (polyallylamine). Interestingly, none of these molecules appeared to be effective in the reduction of resazurin (Figure S18). In contrast, partial redox activity was observed with polyethylenimine (PEI), a poly‐amine polymer (Figure S19a,b). Its activity was attributed to the high density of amines arranged in a branched architecture, which resembles the structural organization of **CDs**, where vicinal amines likely act as anchoring points to promote supramolecular interactions with the substrate. This spatial arrangement brings the two components of the redox couple into close proximity, facilitating the electron transfer. The lower reducing ability of PEI compared to the **CDs** (Figure S19c) can be tentatively explained by the absence of functional groups in PEI other than amines, which in the **CDs** may play a role in the formation of a supramolecular complex with resazurin. In particular, the high density of accessible amino groups arranged in close spatial proximity and anchored to the rigid core of **CDs** appears to be crucial. This configuration enables the assembly of a nano‐supramolecular structure in which **CDs** and resazurin are associated through non‐covalent supramolecular interactions, thereby facilitating electron transfer.

These control experiments, combined with the redox assessments in Figure 3b,c, demonstrated that *(i)* surface amines mediate the redox reaction, *(ii)* small molecular amines are ineffective for this transformation, and *(iii)* polymeric structures with 3D architectures (such as PEI or **CDs**) are required for effective resazurin reduction.

### 2.3 Evaluation of CD/Resazurin Supramolecular Interactions

To study the supramolecular interactions between resazurin and **CDs**, a series of ^1^H‐NMR titration experiments was conducted using **CDs‐1** as the model system. This choice was strategic: **CDs‐1** possess the lowest surface amine density among the three batches, minimizing complications from multiple concurrent binding events and providing the simplest system for accurate quantification of binding parameters. The reduced number of potential interaction sites per nanoparticle facilitates clearer interpretation of NMR chemical shift changes and binding stoichiometry (details are reported in Table S4, Figure S20, and Experimental Section ^1^H‐NMR binding experiments).

**FIGURE 4:**
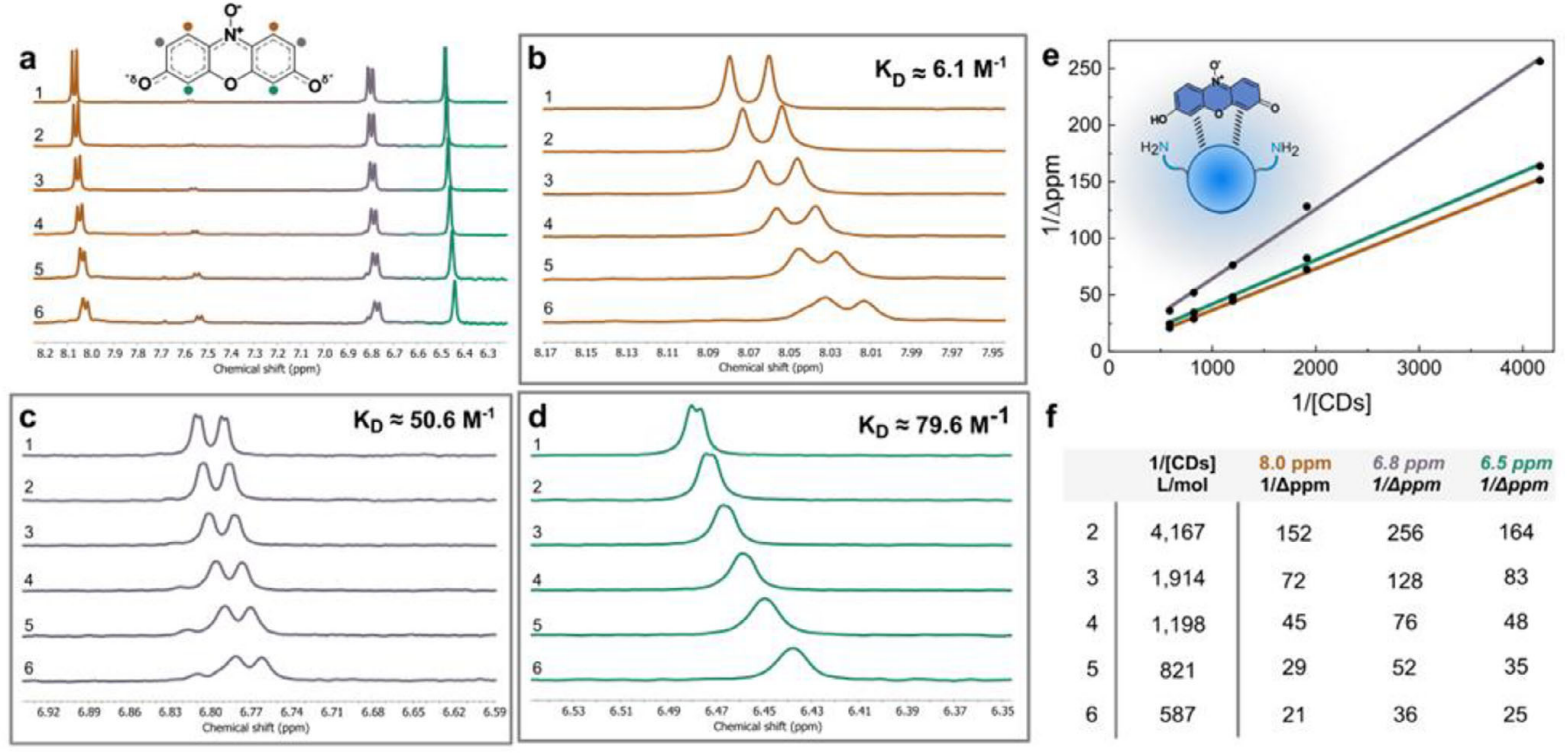
**a)** ^1^H‐NMR spectra acquired in D_2_O carbonate buffer (0.1 M, pH 9.4) of pure resazurin (spectrum 1) and resazurin with increasing **CDs‐1** concentrations from 0 to 1.70 mM (spectra 2‐6), calibrated against residual solvent. **b)** Concentration‐dependent chemical shift of the 8.0 ppm peak from the spectra in **(a)**. **c)** Concentration‐dependent chemical shift of the 6.8 ppm peak from the spectra in **(a)**. **d)** Concentration‐dependent chemical shift of the 6.4 ppm peak from the spectra in **(a)**. **e)** Linear correlation between chemical shifts at 6.4, 6.8, and 8.0 ppm versus reciprocal **CDs** **‐1** concentration. This plot was used to calculate the binding constants (*K_D_*) for **CDs‐1**. **f)** Table reporting the experimental data for the estimation of *K_D_*.

Although resazurin can be represented by a predominant Lewis structure, it exists as a resonance hybrid of multiple contributing forms. This delocalization imparts molecular symmetry, resulting in only three distinct proton signals in the ^1^H‐NMR spectrum at alkaline pH (Figure 4a). Upon incremental addition of **CDs‐1** to the resazurin solution, an upfield shift of all resazurin proton signals was observed (Figure 4b–d). This phenomenon results from **CDs‐1** donating electron density to resazurin, producing a shielding effect that shifts the chemical environment of resazurin protons. The observed signal broadening and loss of multiplicity indicated the formation of supramolecular aggregates between resazurin and **CDs‐1** [17]. In such assemblies, the reduced rotational and diffusional mobility of the protons compared to that of isolated molecules leads them to be exposed to an inhomogeneous magnetic field, which causes the characteristic peak broadening effects [54, 55].

Despite observable broadening and shifting of resazurin ^1^H‐NMR peaks upon **CDs‐1** addition, precise quantification of the supramolecular interaction strength was precluded by concurrent resazurin reduction to resorufin. The increasing intensity of the peak at 7.52 ppm, associated with resorufin protons at 1*H* and 9*H* positions [56], confirmed this reduction (spectra (3)‐(6) in Figure 4a; Figure S20).

Consequently, the maximum **CDs‐1** concentration achievable before resazurin conversion was insufficient to reach binding site saturation required for accurate *K_D_* determination. Nevertheless, *K_D_* was estimated from available data by plotting inverse chemical shift changes (1/Δppm) for each proton signal of resazurin (6.5, 6.8, and 8.0 ppm) against inverse **CDs‐1** concentration (1/[**CDs**]) (Figure 4e,f), with binding constants obtained through linear regression analysis (Equation 16, Experimental Section 1H‐NMR binding experiments) [57]. All *K_D_* values were of similar magnitude, with the 6.5 ppm signal (proximal to hydroxyl/carbonyl groups) exhibiting the highest binding constant (*K_D_* ≈ 79.6 M^−1^). These data suggest hydrogen bond formation between resazurin and **CDs‐1**, with **CD** amine groups serving as donors and resazurin as acceptor, and indicate weak **CD‐**resazurin association.

The supramolecular complex requirement explains why small molecules proved ineffective for resazurin reduction, whereas macromolecules with multiple spatially organized amines (**CDs** and PEI) successfully performed this transformation (Figure S19). Control experiments using formic acid and ethylenediamine as molecular models for **CD** surface carboxylic acids and amines showed no resazurin chemical shift changes (Figure S21), indicating that a critical number of accessible vicinal amines is needed for electron transfer. Since resazurin reduction requires two electrons, at least two vicinal amines are necessary for the reaction to proceed (Figure S5). ITC experiments further evaluated **CDs‐1**/resazurin supramolecular interactions (Experimental Section Isothermal titration calorimetry (ITC) measurements). Titration curve shape can be predicted by the dimensionless parameter *c* = *K_D_* ∙ *X_NM_* (where *X_NM_* is nanomaterial concentration), with well‐defined sigmoidal curves obtained when *c* > 1000 and featureless curves when *c* < 10 [58, 59].

**FIGURE 5:**
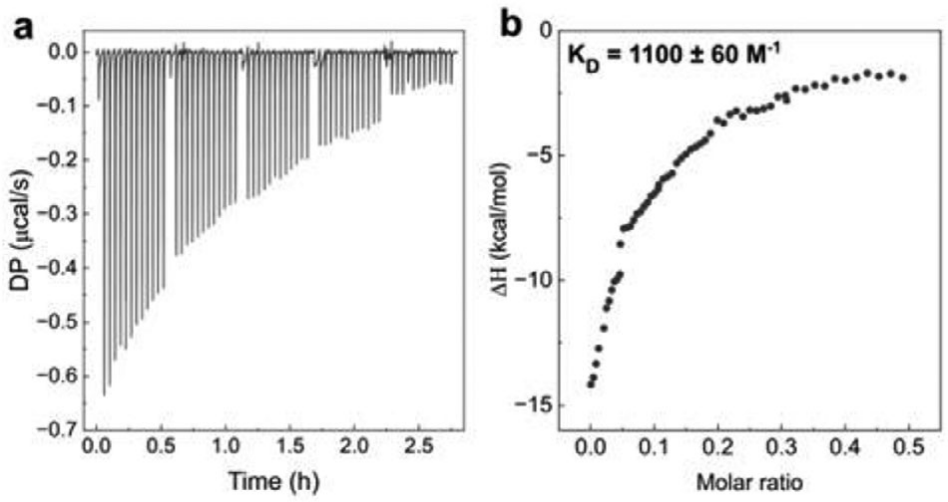
**a)** Plot showing five consequential ITC titration experiments. **b)** Plot showing the integrated ITC data (resazurin/**CD** molar ratio vs enthalpy), and the associated binding constant K_D_. The error on K_D_ was calculated based on the error on K_d_ retrieved from the fitting. See Figure S22, and the Experimental Section isothermal titration calorimetry (ITC) measurements for the details of the fitting. Experiments were performed in carbonate buffer (0.1 m, pH 9.4) with resazurin (2 × 10^−6^ m) and **CDs‐1** (0.8 × 10^−3^ M).

Based on ^1^H‐NMR‐derived low *K_D_* values, ITC titration required experimental modifications. Conventional approaches of increasing ligand and host concentrations are limited by solubility and aggregation issues, as highly concentrated solutions may dissociate endothermically during injection, compromising binding data. Additionally, ITC syringe volume restricts titrant injection amounts. Multiple consecutive titrations overcame these constraints, allowing **CDs‐1**/resazurin molar ratios to increase until the titration curve reached plateau conditions for *K_D_* determination (Figure 5; Figure S22). One‐site binding model fitting of the experimental ITC data according to Equation 17 (Experimental section Isothermal titration calorimetry (ITC) measurements), yielded *K_D_* = 1110 ± 60 m ^−1^. This simplified binding model was appropriate given the low surface amine density of **CDs‐1** (2 ± 1 amines per nanoparticle), suggesting predominant 1:1 stoichiometry between **CDs‐1** and resazurin. This interpretation is supported by the thermodynamic analysis presented below, which indicates formation of two hydrogen bonds per binding event, consistent with two vicinal amines on a single **CDs‐1** particle coordinating one resazurin molecule.

As observed during ^1^H‐NMR titrations, resazurin may undergo reduction upon **CDs‐1** binding during ITC experiments, potentially compromising calorimetric data reliability. Multiple consecutive resazurin injections minimized redox reaction enthalpy contributions to total measured heat by maintaining low resazurin concentrations in the ITC cell after each injection, significantly limiting resorufin formation. Furthermore, the low *K_D_* values from ^1^H‐NMR indicate equilibrium favoring unbound species with minimal complex formation, strongly disfavoring the redox process and rendering reduction‐associated heat release negligible. Consequently, the ITC‐derived *K_D_*, though potentially slightly overestimated, is reliable. The titration curve shape (Figure 5b; Figure S22) provides additional evidence that reduction enthalpy contributions are negligible, as constant heat release from resazurin reduction would produce linear cumulative enthalpy increases without plateau formation, contrary to observed behavior.

Control experiments confirmed no aggregation in ligand or host solutions and negligible heat effects in blank titrations compared to **CDs‐1**/resazurin interactions (Figure S23).

ITC analysis also provided crucial thermodynamic parameters (*ΔH*, *ΔG*, *ΔS*) for the **CDs‐1**/resazurin binding event [22]. The featureless isotherm and retrieved *K_D_* indicate a *c* value (*c* = *K_D_* ∙ [**CDs**]) of approximately 0.9. Under these conditions the binding affinity can be estimated with reasonable accuracy, but the binding enthalpy value is much less reliable unless the binding stoichiometry is precisely known [60]. Therefore, the retrieved *ΔH* represents an estimated enthalpy change for **CDs‐1**/resazurin complexation.

Resazurin binding was thermodynamically favorable (*ΔG* = −4.15 kcal/mol) and predominantly enthalpy‐driven, with enthalpic contribution (*ΔH* = −12.28 kcal/mol) significantly exceeding entropic contribution (*ΔS* = −0.03 kcal/mol). This indicates that favorable non‐covalent interactions (i.e., hydrogen bonding, van der Waals forces, and electrostatic attractions) stabilize the supramolecular complex. These enthalpic contributions compensate for the entropic penalty of reduced resazurin mobility upon complex formation. The *ΔH* value is compatible with two hydrogen bonds between **CDs‐1** and resazurin (single hydrogen bond *ΔH* ≈ 5–8 kcal/mol) [61, 62], suggesting each bond facilitates electron transfer from **CDs‐1** to resazurin.

This study demonstrates one of the first effective applications of ITC for **CD** characterization, providing a quantitative framework for understanding how supramolecular interactions modulate electron transfer efficiency [23, 63, 64]. Both ^1^H‐NMR titration and ITC analyses yielded consistent binding constants, with ITC additionally suggesting 1:1 binding stoichiometry between **CDs‐1** and resazurin through one‐site binding model fitting. The multiple consecutive titration approach offers a valuable strategy for studying low‐affinity systems where conventional methods that rely on concentration increases are precluded by aggregation constraints.

## 3 Conclusions

This work highlights the pivotal role of surface amine groups in modulating the redox and photoredox properties of **CDs**. The synthesis and comparison of three different batches of amine‐rich **CDs** with systematically varied numbers of surface amines and accessibility, evidenced a positive correlation between higher surface amine density and enhanced redox activity under dark and light conditions. Additionally, electrochemical measurements confirmed the thermodynamic feasibility of these processes, yielding a negative variation in Gibbs free energy for both electron transfer and photoinduced electron transfer. Control experiments using small model molecules (aliphatic amines, molecules bearing both aminic and acidic moieties, aromatic amines, and heterocycles) failed to achieve comparable reactivity, indicating that the nanoscale organization and rigid architecture of **CDs** are crucial for stabilizing the substrate‐**CD** complex and enabling efficient electron transfer.

Independent ^1^H‐NMR and ITC experiments further supported these findings, quantifying the binding constant between **CDs** and resazurin and providing the associated thermodynamic parameters of the interaction. Notably, ITC analysis suggested a 1:1 binding stoichiometry between **CDs‐1** and resazurin, consistent with two vicinal surface amines coordinating a single substrate molecule through hydrogen bonding. This molecular‐level understanding of the binding mechanism establishes a foundation for the rational design of host‐guest systems based on **CDs**, enabling predictable control over substrate recognition and electron transfer efficiency. By tailoring the number and spatial arrangement of surface functional groups, it becomes possible to engineer **CD‐**based nanocatalysts with optimized binding affinity and redox activity for specific substrates and transformations.

In this contest, MD‐GPC measurements were pivotal because they enabled estimation of the molecular weight of **CDs** and calculation of the **CD‐**resazurin molar ratio. Moreover, MD‐GPC data allowed for the calculation of the number of surface amine groups per nanoparticle. This was essential to correlate the kinetic data with the actual molar concentration of **CDs** in solution, ensuring a reliable and accurate interpretation of the photoredox abilities of the **CDs**.

These findings not only elucidated the mechanistic aspects of **CD‐**substrate interaction and electron transfer, but also established a valuable method for indirectly probing **CD** surface reactivity through comparative redox efficiency measurements. Looking ahead, the surface‐reactivity investigation framework established in this work paves the way toward the exploitation of different redox substrates for different chemical transformation mechanisms, i.e. hydrogen atom transfer, hydride transfer, or multi‐electron/proton‐coupled processes. This would allow to prove more in depth the role and reactivity of the **CD** surface functional groups. Additionally, using reversible redox probes and substrates with well‐known redox behavior will provide a more quantitative understanding of surface‐mediated reaction pathways. Such investigations, building on the structural and thermodynamic insights reported in this work, represent a promising direction for future studies aimed at further expanding the mechanistic understanding of the complex surface reactivity of **CDs**.

Taken together, these results contribute to the fundamental understanding of the elusive surface chemistry of **CDs**, providing pivotal information for the rational design of the next‐generation of **CD‐**based nanocatalysts. In addition, the 1:1 binding stoichiometry suggested by ITC experiments, and the requirement for multiple vicinal amines open new opportunities for engineering **CDs** with tailored surface functionalities.

By controlling synthetic conditions to achieve specific amine densities and spatial arrangements, it becomes possible to modulate **CD‐**substrate recognition, optimize electron‐transfer kinetics, and expand the application scope of **CDs** across catalysis, sensing, photoredox chemistry, and materials science.

## 4 Experimental Section

### 4.1 Materials

*L*‐Arginine, ethylenediamine,, butylamine, pyridine, polyallylamine (average *M_w_* = 65 000 g/mol), formic acid, 2,6‐pyridinedicarboxylic acid, 2,6‐diaminopyridine, benzylamine, 12‐aminodocecanoic acid, polyethylenimine ethylenediamine end‐capped (PEI, average *M_W_* = 800 g/mol by light scattering, average *M_n_* = 600 g/mol by GPC), resazurin and resorufin sodium salts, Na_2_CO_3_, NaHCO_3_, tetrabutylammonium hexafluorophosphate (TBAPF_6_), NaNO_3_ (EMSURE ACS), and acetic acid (ACS reagent, ≥ 99.8%) were purchased from Sigma–Aldrich. Putrescine was purchased from Alfa Aesar, phenetylamine was purchased from Fluorochem. PolyCAL Pullulan and dextran were purchased from Malvern Panalytical). All the reagents were used as received, without further purification, unless otherwise stated. Anhydrous *N,N*‐dimethylformamide was purchased from Sigma–Aldrich and used without further purification. Flot‐A‐Lyzer dialysis tubes with molecular weight cut‐off 500–1000 Da were purchased from Repligen. Ultrapure fresh water obtained from a Millipore water purification system (>18 MΩ Milli‐Q, Millipore) was used in all experiments. D_2_O was purchased from WVR chemicals. Kessil lamp was purchased from https://kessil.com/products/science_PR160L.php.

### 4.2 Methods

#### 4.2.1 Synthesis of CDs

The **CD** syntheses were performed on a CEM Discover‐SP laboratory microwave following previously published procedures. Briefly *L*‐arginine (87.0 mg, 0.5 mmol, 1 equiv.) or a mixture of *L*‐arginine (0.5 mmol, 1 equiv.) and an alkyl diamine namely ethylenediamine or putrescine (0.5 mmol, 1 equiv.) were solubilized in 100 µL of Milli‐Q water and added into a sealable microwave reaction vessel. The vessel was subsequently heated in a microwave reactor (200 W) at 240°C, 26 bar for 180 s.

After the synthesis, the crude reaction mixtures were diluted to 10 mL with Milli‐Q water, and filtered through a 0.1 µm polytetrafluoroethylene (PTFE) microporous membranes. The solutions were then dialyzed for 48 h against pure Milli‐Q water (MWCO = 500–1000 Da). Finally, the purified products were lyophilized to obtain light yellow solids (See Figure 2a–c). In particular, the synthesis yields were 48%, 25%, and 18% for **CDs 1‐3** respectively [38].

#### 4.2.2 Characterization of CDs

The **CDs** were characterized by ^1^H‐NMR spectroscopy, UV–vis and fluorescence spectroscopy and the results were found to be in good accordance with what previously reported in literature [38].

The ^1^H‐NMR spectra of the purified **CDs** (at least 5 mg of **CDs** were solubilized in 600 µL of solvent) were recorded on Varian 400 spectrometer (^1^H: 400 MHz) using D_2_O as solvent.

The UV–vis spectra for the characterization of the **CDs** were recorded on an Agilent Cary 5000 UV–vis spectrophotometer. Samples were analyzed in Milli‐Q water at the concentration of 10^−1^ mg/mL. After an automatic blank subtraction, all the UV‐Visible spectra were manually zeroed at 800 nm.

Fluorescence spectra for the characterization of **CDs** were recorded in Milli‐Q water on an Edinburgh Instruments FS5 spectrofluorometer (**CDs** concentration = 10^−1^ mg/mL). To acquire the emission maps, the excitation wavelengths were varied from 320 to 440 nm. All the emission spectra were automatically corrected by the instrument using an internal reference file. CD characterization is reported in Figure 2a–e.

#### 4.2.3 Multi‐Detection Gel Permeation Chromatography (MD‐GPC)

Absolute molecular weight and molecular weight distribution of **CDs** was determined by multi‐detection gel permeation chromatography (MD‐GPC) using an Omnisec RESOLVE/REVEAL system (Malvern Panalytical) equipped with refractive index (RI), UV–vis, right angle light scattering (RALS)/low angle light scattering (LALS), and differential viscometer detectors. All data were collected and processed using Omnisec v12 software. The multi‐detector system was calibrated using PolyCAL Pullulan (narrow distribution – calibration, 107 K) and dextran (broad distribution – verification, 68 K) standards. All the mobile phases used were pre‐filtered through a bottle vacuum filter funnel with a 0.22 µm PES membrane (Steritop, Merck). For chromatographic separation of **CDs**, two cationic columns (TSKgel G5000PWXL‐CP + G3000PWXL‐CP, Tosoh Bioscience) were employed with 0.1 M NaNO_3_ at pH 2.6, acidified with acetic acid (*ca*. 0.5% v/v) mobile phase at a 0.5 mL/min of flow rate (column and detector oven kept at 20°C, whereas autosampler at 8°C). Samples were prepared at a concentration of *ca*. 5 mg/mL, dissolved in the eluent, and filtered through 0.22 µm nylon syringe filters. CD MD‐GPC characterization is reported in Figure 2f,g, Figures S1–S3 and Table S1.

The absolute molecular weight and molecular weight distribution of **CDs 1‐3** and their physical‐chemical properties (i.e., *dA/dC* variation of sample absorbance with the concentration, *dn/dC* variation of sample refractive index with the concentration, and *R_H_* hydrodynamic radius) were determined using our previously established procedure [25]. Briefly, in MD‐GPC, accurate analysis requires reliable concentration values for *dn/dC* and *dA/dC* calibration. However, the RI chromatograms of **CDs 1‐3** displayed a broad elution band at 17.8–18.9 mL, partially overlapping with a sharp permeation peak at 20.5–21.5 mL attributable to low‐molecular‐weight impurities (Figures S1–S3) [25]. Such peak overlap introduces concentration errors and leads to systematic underestimation of *dn/dC*. To overcome this issue, we employed the UV–vis detector at 350 nm, where only the **CD** component absorbs. Full integration of the UV trace at 350 nm yielded the **CD** concentration (excluding impurities) and the corresponding *dA_350_/dC* parameter. This corrected concentration value was then applied to the RI signal, allowing extraction of *dn/dC* solely from the main **CD** peak while excluding the impurity contribution. With the experimentally determined *dn/dC*, and the light scattering and viscometer signals, the absolute number‐average (*M_n_*) and weight‐average (*M_w_*) molecular weights, dispersity (*Ð*), and hydrodynamic radii (*R_H_*) of **CDs 1‐3** were obtained (Table S1).

#### 4.2.4 Dynamic Light Scattering (DLS)

DLS measurements were performed on a Zetasizer Nano ZS (Malvern Panalytical Ltd) using a Zeta‐Potential cuvette (Malvern Panalytical Ltd, model DTS1070). **CD** samples were prepared at a concentration of 4 mg/mL in the GPC buffer (0.1 m NaNO_3_ + 0.5 v/v% acetic acid pH 2.6). Before each measurement, samples were filtered through a 0.22 µm nylon syringe filter. Resulting plots are reported in Figure S4.

#### 4.2.5 Reduction of Resazurin

For this work, the reduction of resazurin was conducted at room temperature in carbonate buffer (0.1 M, pH 9.4 or 10.8) in a semi‐micro polymethylmethacrylate (PMMA) cuvette (path‐length 1 cm, final volume 1 mL). The experiments were carried out with a resazurin concentration of 2 × 10^−6^ m and by varying the concentration of **CDs** as described in the text.

For the experiments in the dark (Figure S7a), the cuvette with **CDs** and resazurin was placed inside an Agilent Cary 3500 Multicell Peltier UV–vis spectrophotometer, and the reaction was automatically monitored for 26 min by acquiring a spectrum every 2 min. After an automatic blank subtraction, all the UV‐Visible spectra were manually zeroed at 800 nm. Numerical data was analyzed using Microsoft Excel and OriginLab software, and plotted using OriginLab.

For the experiments under light irradiation (Figure S7b), the cuvette with **CDs** and resazurin was placed under a Kessil lamp (*λ_max_* = 427 nm, 45 W, Irradiance = 200 mW/cm^2^) at its maximum power, and in the absence of band filters. The wavelength of the light source was chosen according to the absorption of **CDs** and resazurin, in order to efficiently excite the **CDs** without degrading the resazurin (*λ_max_* = 600 nm) or the resorufin product (*λ_max_* = 570 nm). For the experiments under light irradiation (Figure S7b), the cuvette with **CDs** and resazurin was placed under a Kessil lamp (*λ_max_* = 427 nm, 45 W, Irradiance = 200 mW/cm^2^) at its maximum power, and in the absence of band filters. The wavelength of the light source was chosen according to the absorption of **CDs** and resazurin, in order to efficiently excite the **CDs** without degrading the resazurin (*λ_max_* = 600 nm) or the resorufin product (*λ_max_* = 570 nm). In fact, as reported in Table 1, **CDs 1‐3** present an absorption tail up to ≈450 nm (**CDs‐1** *E_00_* = 2.72 eV = 455 nm; **CDs‐2** *E_00_* = 2.77 eV = 447 nm; **CDs‐3** *E_00_* = 2.82 eV = 439 nm). Moreover, the Kessil lamp employed for the photoredox assessments is not a monochromatic light source, and its light emission presents a Gaussian shape with emission in the 390–470 nm range (full width at half maximum), which well overlaps with the absorption spectra of all types of **CDs**.

The reaction was monitored using an Agilent Cary 3500 Multicell Peltier UV–vis spectrophotometer for 25 min by manually acquiring a spectrum every 5 min. After an automatic blank subtraction, all the UV‐Visible spectra were manually zeroed at 800 nm. Numerical data was analyzed using Microsoft Excel and OriginLab software, and plotted using OriginLab.

The resazurin consumption and resorufin production were calculated starting from the experimental data according to the following equations:

(1) $$A_{570}=\varepsilon_{resazurin_{570nm}}\cdot l\cdot C_{resazurin}+\varepsilon_{resorufin_{570nm}}\cdot l\cdot C_{resorufin}$$

(2) $$A_{600}=\varepsilon_{resazurin_{600nm}}\cdot l\cdot C_{resazurin}+\varepsilon_{resorufin_{600nm}}\cdot l\cdot C_{resorufin}$$

where *l* is the optical path length (equal to 1 cm), *C* is the concentration of the species involved (in mol/L) in the reaction, and ε is the molar excitation coefficient in water at the specified wavelength. The molar extinction coefficients used for the calculation are reported in Table S2 [65]. The results of the (photo)redox assessments are reported in Figure 3.

#### 4.2.6 Quantum Yield (QY) Estimation

In a typical photo‐reaction an aqueous solution of **CDs‐1** (4 mg/mL) and resazurin (2 × 10^−6^ m) in carbonate buffer (0.1 m, pH 9.4) was continuously irradiated with a Kessil lamp (*λ_max_* = 427 nm, 45 W, Irradiance = 200 mW/cm^2^). The QY was estimated according to the following equations [66].

First, the energy of a photon can be obtained from Equation 3.

(3) $$E\left(J\right)=\frac{h\;\cdot c\;}{\lambda }$$

Where *h* is the Planck's constant (6.626 × 10^−34^ J∙s), *c* is the speed of light 3 × 10^8^ m/s, and *λ* is the wavelength at the maximum intensity of the lamp (4.27 × 10^−7^ m).

Then, the moles of photons per second can be derived according to Equation 4.

(4) $$mol\;photon/s=\frac{P}{E\cdot NA}=\frac{P\cdot \lambda }{h\cdot c\cdot NA}=1.61\cdot 10^{-4}\;mol/s$$

Where P = 45 W = 45 J/s and NA is the Avogadro's number = 6.022 × 10^23^ mol^−1^. Once the number of photons per second was obtained, this was multiplied by the reaction times reported in Table S3 to determine the total number of incident photons at any given time. These moles of photons were then correlated with the moles of product (resorufin), as reported in Figure S13. The amount of resorufin produced for each time point was then determined from the linear fit of the experimental kinetic data of **CDs‐1** mediated resazurin reduction.

#### 4.2.7 CDs‐1 Reusability

To test the reusability of **CDs‐1**, the reaction mixture of a standard redox experiment (carbonate buffer 0.1 M, pH 9.4, [**CDs‐1**] = 4 mg/mL, [resazurin] = 2 × 10^−6^ M) was recovered after the reaction. To remove the traces of unreacted resazurin and of the resorufin product, the reaction mixture was then dialyzed for 48 h against pure Milli‐Q water (MWCO = 500–1000 Da). After dialysis, the **CDs** were lyophilized and analyzed with UV–vis spectrometry. Results are reported in Figure S14.

#### 4.2.8 Zeta‐Potential Measurements

Zeta potential measurements were performed on a Zetasizer Nano ZS (Malvern Panalytical Ltd) using a Zeta‐Potential cuvette (Malvern Panalytical Ltd, model DTS1070). **CD** samples were prepared at a concentration of 4 mg/mL in carbonate buffer (0.005 M, pH 9.4). Before each measurement, samples were filtered through a 0.22 µm nylon syringe filter. Resulting plots are reported in Figure S15.

#### 4.2.9 Cyclic Voltammetry

Cyclic voltammetry was carried out at room temperature on a saturated solution of **CDs** in *N,N*‐dimethylformamide (DMF) with 0.1 M of tetrabutylammonium hexafluorophosphate (TBAPF_6_), following an already published procedure [51]. Briefly, the measurements were conducted in a three‐electrode electrochemical cell using glassy carbon electrodes as the working electrode, a platinum wire as counter electrode, and a saturated calomel electrode (SCE) as the reference electrode. Oxygen was removed by purging the analyte solution with argon for *ca*. 2 min. Cyclic voltammetry was carried out with a scan rate of 100 mV/s. Numerical data was plotted using OriginLab software.

Based on the numerical results retrieved from cyclic voltammetry (Figure S16), the excited state potential of **CDs** E*( ***CDs*** *^+^/* ***CDs*** *) was calculated by means of the Rehm–Veller equation:

(5) $$E^{*}\left(CDs/CDs^{*}\right)=E^{OX}\left(CDs/CDs^{+}\right)-E_{00}\left(CDs/CDs^{*}\right)$$

where *E^OX^ (* ***CDs*** */* ***CDs*** *^+^)* is the ground state redox potential of **CDs** obtained from cyclovoltammetry, and *E_00_* (**CDs**/CDs*) is their excited state energy. *E_00_* (**CDs**/CDs*) may be estimated spectrophotometrically from the position of the short wavelength tail of the absorbance spectrum of the **CDs** (446, 448, and 440 nm for **CDs 1‐3**, respectively), which translates into a *E_00_* of 2.72, 2.77, and 2.82 eV, respectively. (See Table 1; Figure S17).

The Gibbs free energy of the first electron transfer (*ΔG_ET_*) and photoinduced electron transfer (*ΔG_PET_*) between **CDs** and resazurin were calculated according to the following questions:

(6) $$\Delta G_{ET}=F\cdot \Delta E$$

(7) $$\Delta E=E^{RED}\left(X/X^{+}\right)-E^{RED}\left(X/X^{-}\right)+C$$

(8) $$\Delta G_{PET}=F\cdot \Delta E^{*}$$

(9) $$\Delta E^{*}=E^{*}\left(X^{+}/X^{*}\right)-E^{RED}\left(X/X^{-}\right)+C$$

where F is the Faraday constant (23.06 kcal/(V mol)), *C* a coulombic factor dependent on the solvent that can be neglected, *E^RED^* (X^+^/X) is the potential of **CDs** retrieved by cyclic voltammetry, and *E^RED^* is the reduction potential of resazurin (−0.29 V *vs* SCE) [52]. It is worth mentioning that in order to apply Equation 6, the potential of **CDs** must be considered as a reduction, and therefore indicated as a negative value.

(10) $$\begin{array}{ccc} \Delta G_{ET\;\left(CDs-1\right)} & = & 23.06\;\frac{kcal}{V\cdot \mathrm{mol}}\;\cdot \left[-1.27\;V-\left(-0.29\;V\right)\right]\\ & = & -22.60\frac{kcal}{mol} \end{array}$$

(11) $$\begin{array}{ccc} \Delta G_{ET\;\left(CDs-2\right)} & = & 23.06\;\frac{kcal}{V\cdot \mathrm{mol}}\;\cdot \left[-1.30\;V-\left(-0.29\;V\right)\right]\\ & = & -23.29\frac{kcal}{mol} \end{array}$$

(12) $$\begin{array}{ccc} \Delta G_{ET\;\left(CDs-3\right)} & = & 23.06\;\frac{kcal}{V\cdot \mathrm{mol}}\;\cdot \left[-1.34\;V-\left(-0.29\;V\right)\right]\\ & = & -24.21\frac{kcal}{mol} \end{array}$$

According to Equation 8, *ΔG_PET_* was also calculated.

(13) $$\begin{array}{ccc} \Delta G_{PET\;\left(CDs-1\right)} & = & 23.06\;\frac{kcal}{V\cdot \mathrm{mol}}\cdot \left[-1.45\;V-\left(-0.29\;V\right)\right]\\ & = & -26.75\frac{kcal}{mol} \end{array}$$

(14) $$\begin{array}{ccc} \Delta G_{PET\left(CDs-2\right)} & = & 23.06\;\frac{kcal}{V\cdot \mathrm{mol}}\;\cdot \left[-1.47\;V-\left(-0.29\;V\right)\right]\\ & = & -27.21\frac{kcal}{mol} \end{array}$$

(15) $$\begin{array}{ccc} \Delta G_{PET\;\left(CDs-3\right)} & = & 23.06\;\frac{kcal}{V\cdot \mathrm{mol}}\;\cdot \left[-1.48\;V-\left(-0.29\;V\right)\right]\\ & = & -27.44\frac{kcal}{mol} \end{array}$$

#### 4.2.10 ^1^H‐NMR Binding Experiments

To calculate the binding constant *K_D_* between **CDs‐1** and resazurin, several ^1^H‐NMR spectra of resazurin with increasing concentration of **CDs** were recorded in D_2_O carbonate buffer 0.1 m at pH 9.4. The total volume of the solution was 0.8 mL. The concentration of resazurin was kept constant at 6 × 10^−6^ M and the amount of **CDs‐1** was increased according to Table S3. The ^1^H‐NMR binding experiments were conducted on a Varian 400 spectrometer (^1^H: 400 MHz). The spectra were analyzed and plotted using MestreNova – MestreLab software. Binding constant *K_D_* was retried using the double reciprocal method following an already published procedure [57].

The binding constant *K_D_* was calculated by the linear fitting of the data reported in Figure 4e,f according to the following equation [57]:

(16) $$K_{D}=\frac{intercept}{slope}$$

#### 4.2.11 Isothermal Titration Calorimetry (ITC) Measurements

ITC measurements were performed at constant atmospheric pressure and at a constant temperature of 298 K, using an ultrasensitive MicroCal PEAQ‐ITC microcalorimeter (Malvern, UK). The **CDs‐1** (host, 0.8 × 10^−3^ m, 4 mg/mL) and the resazurin (titrant, 2 × 10^−6^ M) were solubilized in carbonate buffer (0.1 M, pH 9.4). The **CDs** solution was placed in the cell of the calorimeter, and the resazurin solution (titrant) was loaded into the injection syringe. To obtain a saturation curve for the **CD**/resazurin binding, five consecutive experiments were performed. During each experiment, after the initial injection of 0.4 µL, 12 injections of 3 µL of titrant were performed with a spacing of 150 s (injection duration 6 s). The reference power was set at 10 μcal/s, the stirrer speed was kept constant at 750 rpm, and the temperature was set at 298 K. The titration data were plotted and analyzed using the Originlab software.

Experimental data and fitting results are shown in Figure S22. *K_D_* was determined by fitting to a one‐site binding model (Equation 17), assuming 1:1 binding stoichiometry [67].

(17) $$y=\frac{B_{max}\cdot x}{K_{d}+x}+y_{0}$$

Where *B_max_* (−1.89 kcal/mol) represents the asymptotic minimum of *ΔH* variation, corresponding to the point where all binding sites are saturated (at which *ΔH* is 0 kcal/mol); *K_d_* is the dissociation constant (defined as 1/*K_D_*); *y_0_* refers to the enthalpy variation after the first binding (−14.18 kcal/mol); *x* refers to the concentration of resazurin during the titration, as reported on the *x* axis of Figure S22); and *y* is the fitted Δ*H* value. After the fitting of the raw data, *K_D_* was calculated as 1/*K_d_*.

The binding enthalpy *ΔH* was retrieved as the difference in enthalpy between the first and the last injection point, yielding a value of −12.28 kcal/mol. *ΔG* and *ΔS* were then calculated according to Equations 18 and 19.

(18) $$\Delta G=-RT\cdot ln\left(K_{D}\right)=-4.15\;kcal/mol$$

Were *R* = 1.987 cal/(mol K); *T* = 298 K; and *K_D_* = 1110 M^−1^

(19) $$\Delta S=\frac{\Delta H-\Delta G}{T}=0.03\;kcal/mol$$

Were *ΔH = −*12.28 kcal/mol; *ΔG = −4.15 kcal/mol*; and *T* = 298 K.

#### 4.2.12 Statistical Analysis

Statistical analysis of the experimental data, including pre‐processing of data, are described within the relevant figure captions throughout the manuscript. Statistical analyses were conducted to all the data retrieved from the experiments, and any identified outliers were retained and incorporated into the overall variability, as reflected in the reported standard deviations. Unless explicitly stated otherwise within a figure or experiment description, numerical experimental results are reported as the mean values ± standard deviation and are derived from experiments performed in triplicate (n = 3). Statistical analyses were conducted using Microsoft Excel and OriginLab software, and final data representations were obtained using OriginLab.

## Funding

The authors gratefully acknowledge the financial support from the European Research Council (ERC AdG‐2019 n.885323, e‐DOTS; ERC StG n. 101039578, PROTOMAT), the University of Trieste, INSTM, and the Italian Ministry of University MUR (cofin Prot. 20228YFRNL). G.F. kindly acknowledges FRA2024 funded by the University of Trieste and Microgrants 2025 funded by Region FVG (LR 2/2011, ART. 4).

## Conflicts of Interest

The authors declare no conflicts of interest.

## Supporting information




**Supporting File**: smll72998‐sup‐0001‐SuppMat.docx.

## Data Availability

The data that support the findings of this study are available in the supplementary material of this article.
