# Supplementary material for: Going Deep into the Surface Chemistry of Carbon Dots: Influence of Functional Groups on the Redox Abilities
Source: Small. 2026 Mar 3;22(24):e14420. doi: 10.1002/smll.202514420 (PMC13114505; doi:10.1002/smll.202514420)
Supplement: Supplementary file 1 — Supporting File: smll72998‐sup‐0001‐SuppMat.docx. [file SMLL-22-e14420-s001.docx]

Supporting information for

Going Deep into the Surface Chemistry of Carbon Dots: Influence of Functional Groups on the Redox Abilities

Summary

[1.1 Multi-detection gel permeation chromatography (MD-GPC) 2](#_Toc220875100)

[1.2 Dynamic light scattering (DLS) 3](#_Toc220875101)

[1.3 Reduction of resazurin 3](#_Toc220875102)

[1.3.1 CDs-1 mediated reduction of resazurin in sub-stoichiometric loadings (pH 9.4) 5](#_Toc220875103)

[1.3.2 Stability of resazurin, resorufin and CDs in carbonate buffer 5](#_Toc220875104)

[1.3.3 CDs-1 mediated reduction of resazurin (in the dark, pH 9.4) 7](#_Toc220875105)

[1.3.4 CDs-1 mediated reduction of resazurin (in the dark, pH 10.8) 8](#_Toc220875106)

[1.3.5 CDs-1 mediated reduction of resazurin (under light irradiation, pH 9.4) 9](#_Toc220875107)

[1.4 Quantum yield (QY) estimation 10](#_Toc220875108)

[1.5 CDs-1 reusability 11](#_Toc220875109)

[1.6 Zeta-potential measurements of CDs 1-3 11](#_Toc220875110)

[1.7 Cyclic voltammetry 11](#_Toc220875111)

[1.8 Control experiments with molecular models 12](#_Toc220875112)

[1.9 ^1^H-NMR binding experiments 14](#_Toc220875113)

[1.10 Isothermal titration calorimetry (ITC) measurements 15](#_Toc220875114)

[Bibliography 16](#_Toc220875115)

## Multi-detection gel permeation chromatography (MD-GPC)


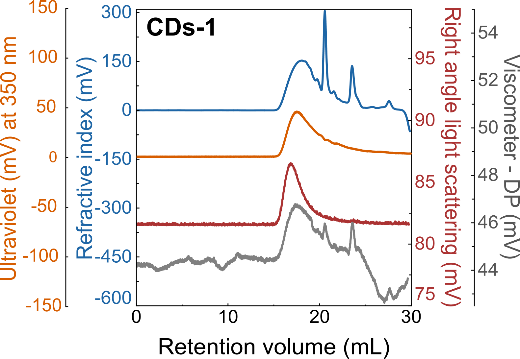


**Figure S1.** MD-GPC chromatograms of **CDs-1** (sample concentration = 5 mg/mL, 0.1 M NaNO_3_ + 0.5 v/v% acetic acid (pH 2.6) on cationic columns). From top to bottom the chromatograms are: refractive index detector response (blue plot), UV detector response at 350 nm (orange plot), light scattering detector response (red plot), and viscometer detector response (grey plot).


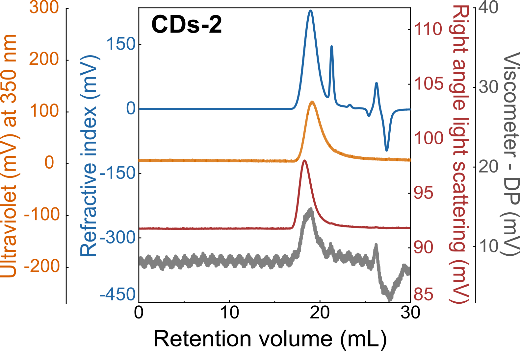


**Figure S2.** MD-GPC chromatograms of **CDs-2** (sample concentration = 5 mg/mL, 0.1 M NaNO_3_ + 0.5 v/v% acetic acid (pH 2.6) on cationic columns). From top to bottom the chromatograms are: refractive index detector response (blue plot), UV detector response at 350 nm (orange plot), light scattering detector response (red plot), and viscometer detector response (grey plot).


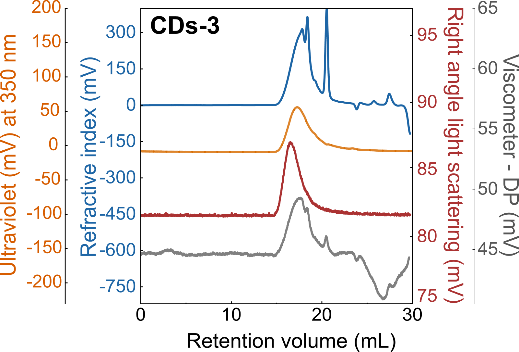


**Figure S3.** MD-GPC chromatograms of **CDs-3** (sample concentration = 5 mg/mL, 0.1 M NaNO_3_ + 0.5 v/v% acetic acid (pH 2.6) on cationic columns). From top to bottom the chromatograms are: refractive index detector response (blue plot), UV detector response at 350 nm (orange plot), light scattering detector response (red plot), and viscometer detector response (grey plot).

As reported in **Table S1**, **CDs-2** and **CDs-3** were the purest, with a recovery rate over 90%. In contrast, **CDs-1** exhibited a higher level of molecular impurities, despite the careful purification protocol applied. This may indicate that using alkyl diamines as the starting materials not only increased the amount of amines on the surface of **CDs**, but also drove their synthesis towards the desired product. This reduced molecular fluorophore content and facilitated obtaining pure samples.

***
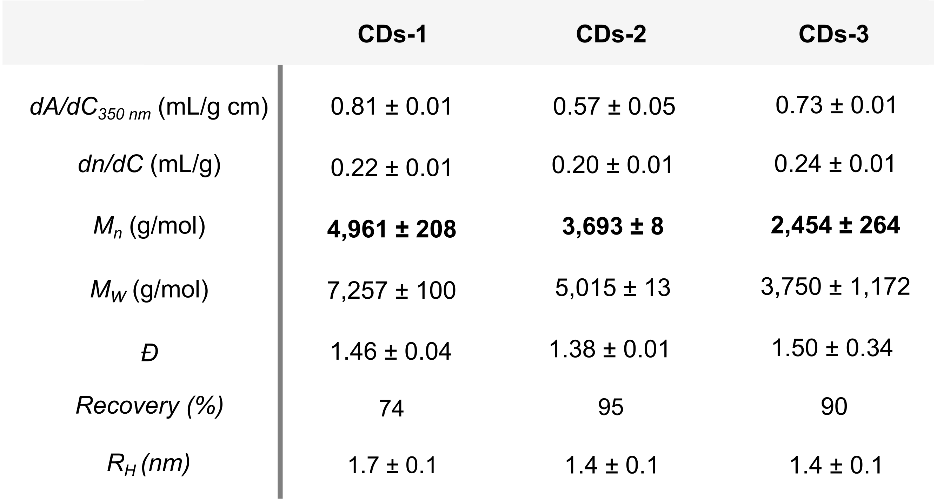
***

***Table S1.*** *Table summarizing the MD-GPC results.*

## Dynamic light scattering (DLS)

As shown in **Figure S4**, aggregates were identified in all the samples analyzed. Interestingly, the intensity of these aggregates decreases from **CDs-1** to **CDs-3**. This is consistent with the increased number of amines on the surface of **CDs**, which are protonated in acidic solutions and facilitate the solubilization of the **CD** samples.


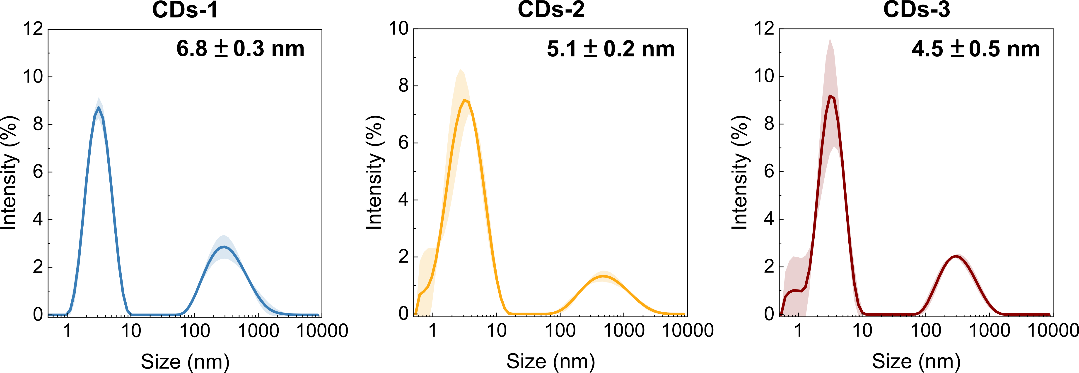


**Figure S4.** Size distribution by intensity of **CDs 1-3** (4 mg/mL, 0.1 M NaNO_3_ + 0.5 v/v% acetic acid, pH 2.6). The average size of **CDs** is indicated in the figure. All the spectra reported the presence of aggregates with big dimensions (>100 nm). Errors bands refer to the standard deviation over three replicas.

## Reduction of resazurin

Resazurin is a purple blue-colored dye that can undergo two reductions: first, an irreversible reduction to resorufin, followed by a reversible reduction to dihydroresorufin. This work focuses on the initial irreversible transformation to resorufin, which serves as a useful indicator of the redox abilities of the tested reducing agents, *i.e.* **CDs**.

The reduction of resazurin to resorufin can follow different reaction pathways (**Figure S5**). These pathways may depend on the reaction pH and on the reducing agent employed.^1^ Previous studies based on cyclic voltammetry showed that the reduction of resazurin to resorufin in aqueous solutions takes place with two one-electron processes.^2^


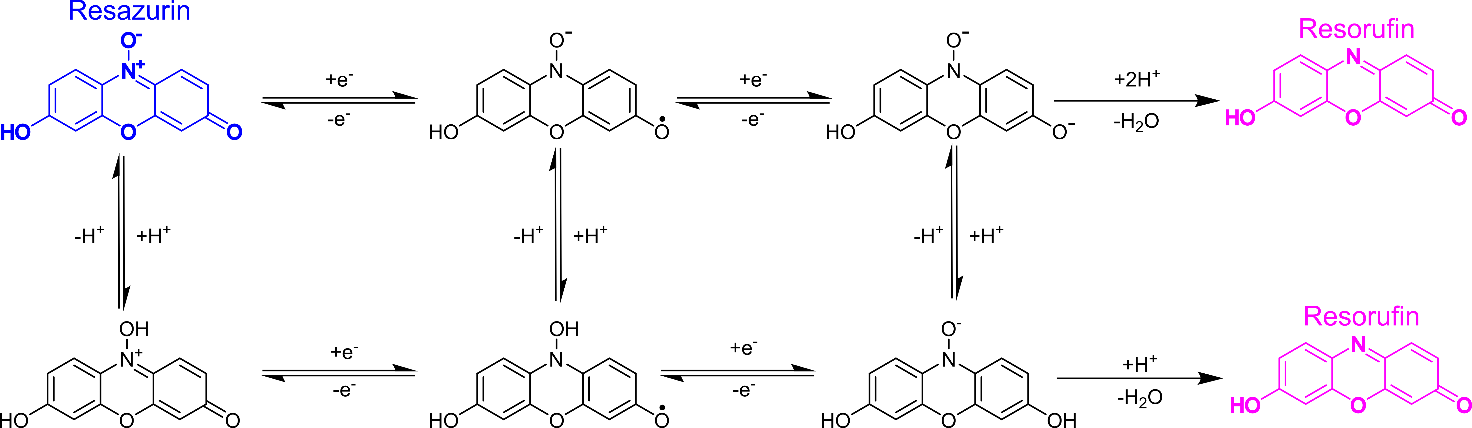


**Figure S5.** Possible reaction pathways for resazurin reduction.

As represented in **Figure S6**, in our system, the electron may be given by the **CDs**, specifically by the amino groups on their surface.


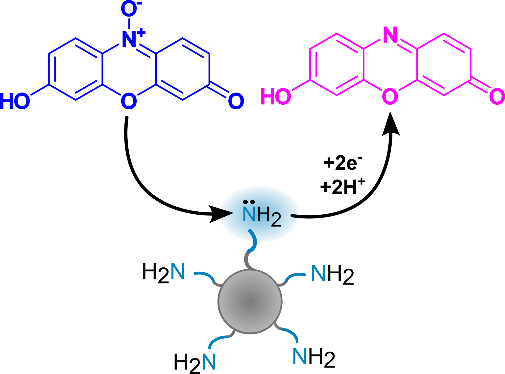


**Figure S6.** Schematic representation of the possible mechanism for the reduction of resazurin to resorufin.


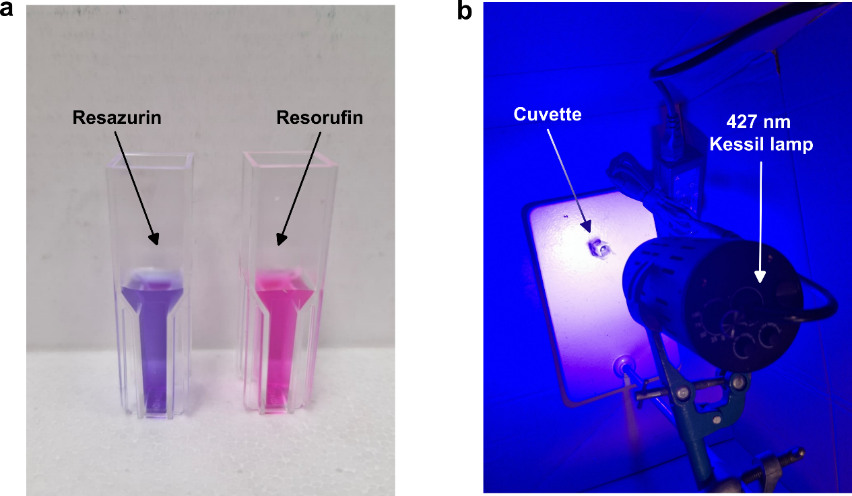


**Figure S7.** **a)** Cuvettes containing resazurin and resorufin in carbonate buffer (0.1 M, pH 9.4), at a concentration 2x10^-6^ M. **b)** Experimental setup for the reduction of resazurin carried out under light irradiation. A Kessil lamp (λ_max_ = 427 nm, 45 W, Irradiance = 200 mW/cm^2^) was used as the visible light source at its maximum power, and in the absence of band filters.


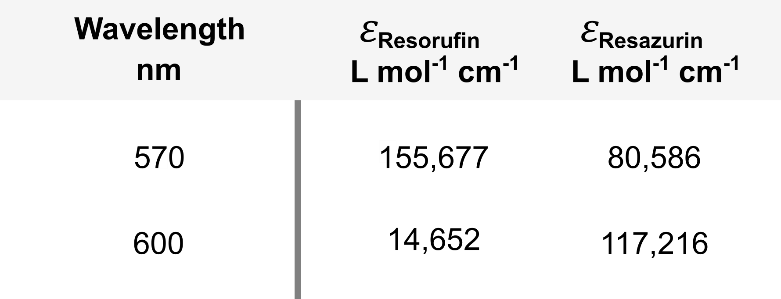


**Table S2.** Table summarizing the molar extinction coefficients ($\varepsilon$) for resazurin and resorufin at the two specified wavelengths.

### CDs-1 mediated reduction of resazurin in sub-stoichiometric loadings (pH 9.4)

When the photoredox abilities of **CDs-1** were tested in catalytic and sub-stoichiometric loadings, resazurin reduction was found to proceed slowly, requiring up to several days for completion under dark conditions. On the other hand, under light irradiation, significant resorufin photobleaching occurs. For these reasons, the (photo)redox abilities of **CDs 1-3** were tested with a large excess of **CDs** compared to resazurin.


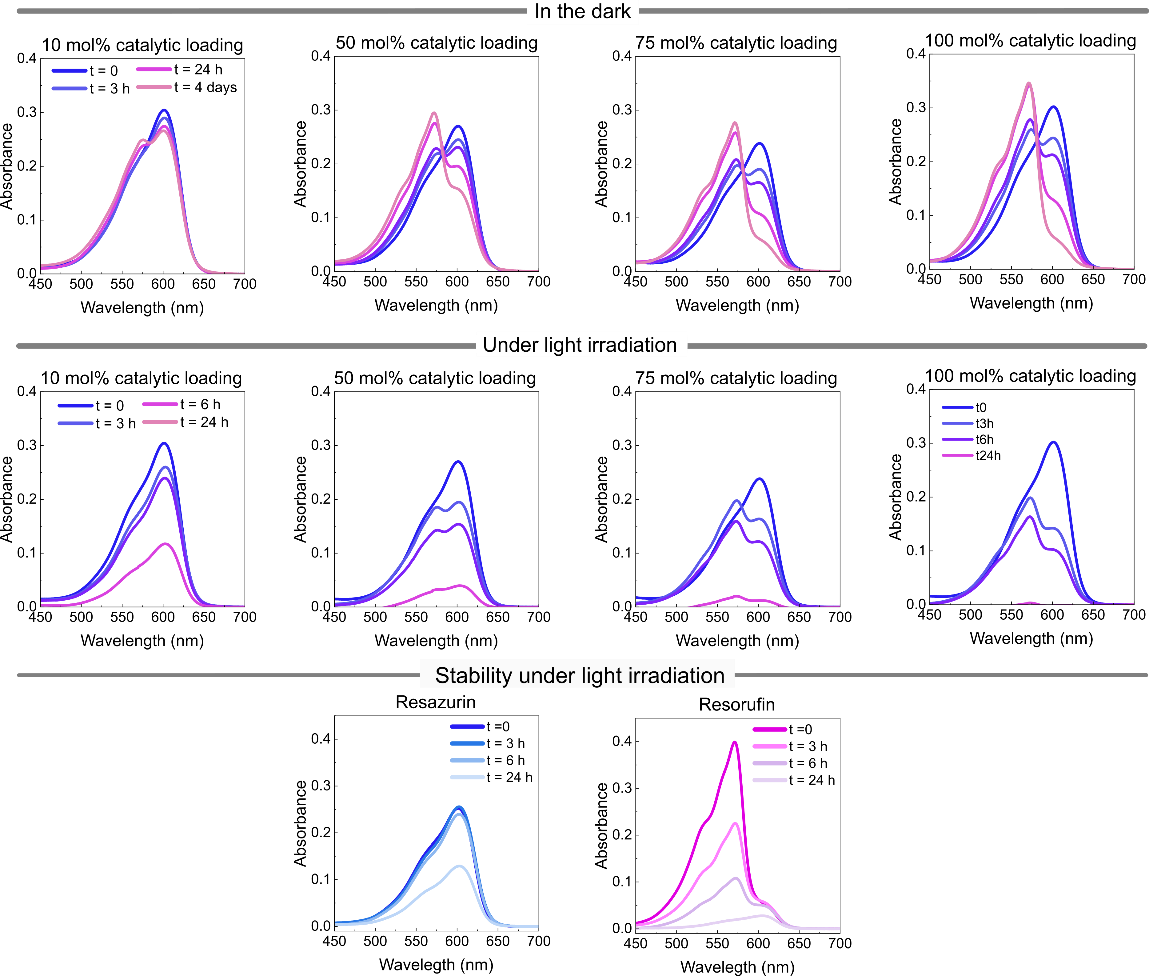


**Figure S8.** **CDs-1** mediated reduction of resazurin in the dark and under light irradiation at pH 9.4. To carry out the experiments the amount of **CDs-1** was reduced up to 10 mol%. For the experiments in the dark, a solution containing **CDs-1** and resazurin in carbonate buffer (0.1 M, pH 9.4) was placed in a PMMA semi-micro cuvette (reaction volume = 1 mL), and the reaction was monitored for 4 days by UV-Vis spectrophotometry by manually acquiring a spectrum at the time points indicated in the figure. For the experiments under light irradiation, a Kessil lamp (λ_max_ = 427 nm, 45 W, Irradiance = 200 mW/cm^2^) was used as visible light source at its maximum power, and in the absence of band filters. The reaction was monitored for 24 h by UV-Vis spectrophotometry by manually acquiring a spectrum at the time points indicated in the figure. For both the sets of experiments resazurin concentration was kept stable at 2x10⁻⁶ M.

### Stability of resazurin, resorufin and CDs in carbonate buffer

To increase the rate of resazurin reduction and to minimize the degradation of resorufin, **CDs 1-3** were tested with a concentration spanning from 1 to 6 mg/mL and the reaction kinetics were carried for 25 min both in the dark and under light irradiation. Before performing the redox experiments, the stability of resazurin, resorufin and **CDs** in carbonate buffer 0.1 M at pH 9.4 or 10.8 was examined both in the dark and under irradiation. Experimental details and outcome are reported in **Figure S9**. All the reagents appeared to be stable in the dark, although a slight degradation of all reagents was observed under light irradiation.


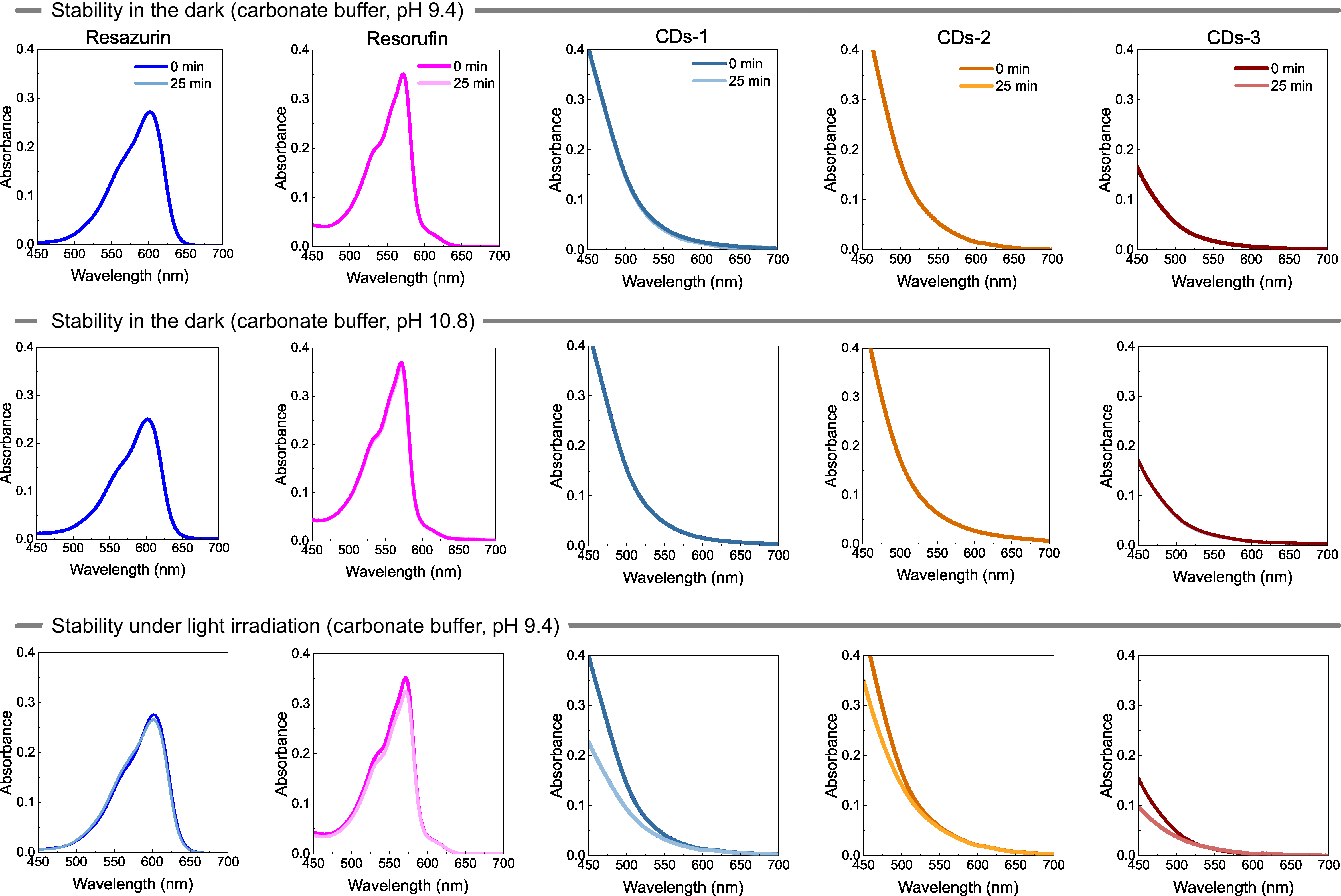


**Figure S9.** Stability of resazurin, resorufin and **CDs 1-3** in the dark and under light irradiation. The stability assessments were carried out in carbonate buffer 0.1 M (pH is indicated in the figure) for 25 minutes. Resazurin and resorufin concentration: 2x10^-6^ M, **CDs** concentration 4 mg/mL. A Kessil lamp (λ_max_ = 427 nm, 45 W, Irradiance = 200 mW/cm^2^) was used as visible light source at its maximum power, and in the absence of band filters.

### CDs-1 mediated reduction of resazurin (in the dark, pH 9.4)


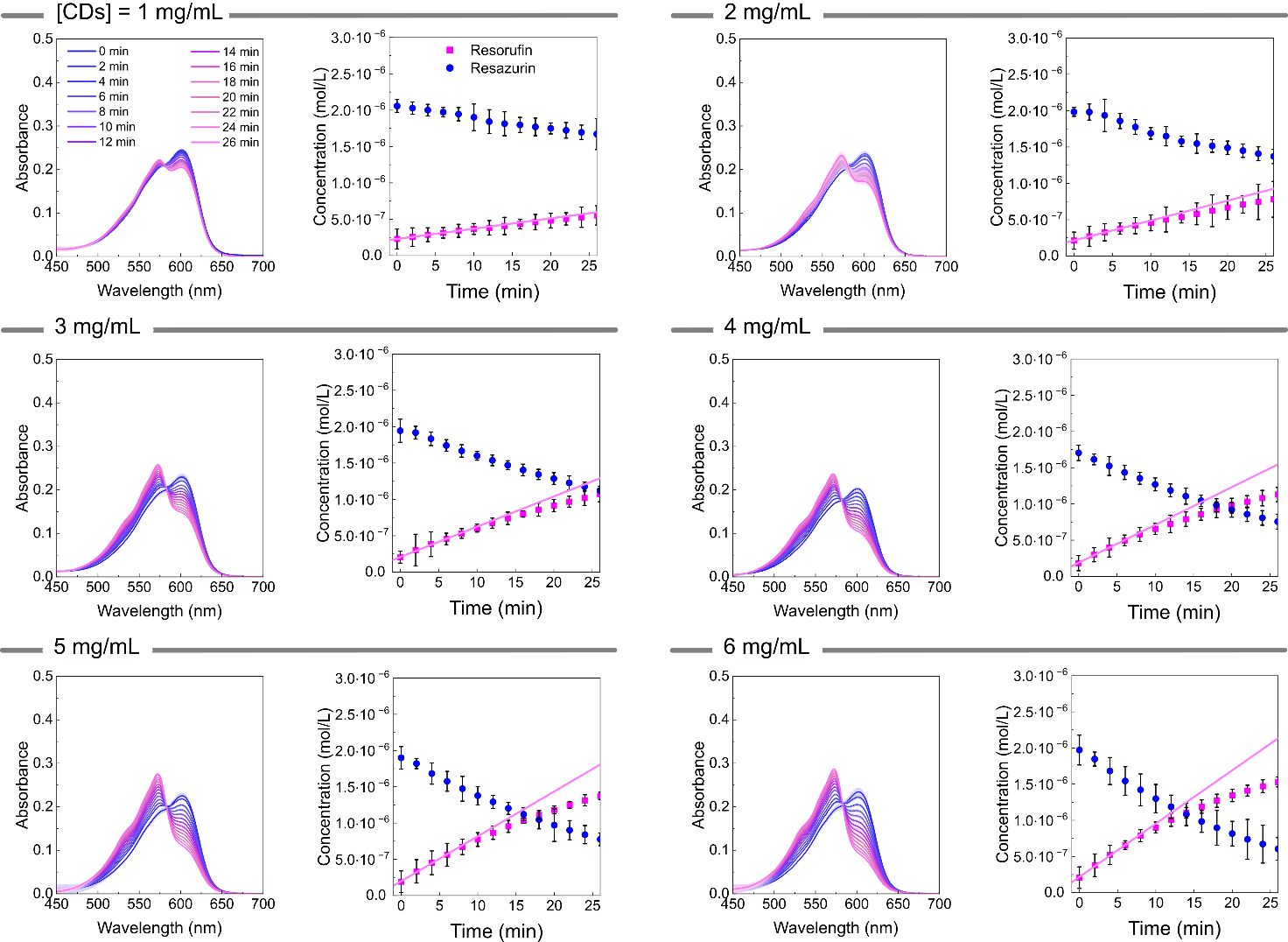


**Figure S10.** **CDs-1** mediated reduction of resazurin in the dark at pH 9.4. A solution containing **CDs-1** and resazurin in carbonate buffer (0.1 M, pH 9.4) was placed in a PMMA semi-micro cuvette (reaction volume = 1 mL), and the reaction was automatically monitored for 26 min by UV-Vis spectrophotometry, acquiring a spectrum every 2 min. The resazurin concentration was kept stable at 2x10⁻⁶ M, while the **CDs-1** concentration was varied as indicated in the figure. The graphs reported are the time-dependent UV-Vis spectra acquired by the instrument and the derived plots of time-dependent changes in resazurin and resorufin concentration. Derived data were obtained according to **Equation 1** and **Equation 2**. The light pink lines reported in the graph refer to the linear fitting for the initial velocity calculation. Error bars and error bands refer to the standard deviation over three replicas.

### CDs-1 mediated reduction of resazurin (in the dark, pH 10.8)


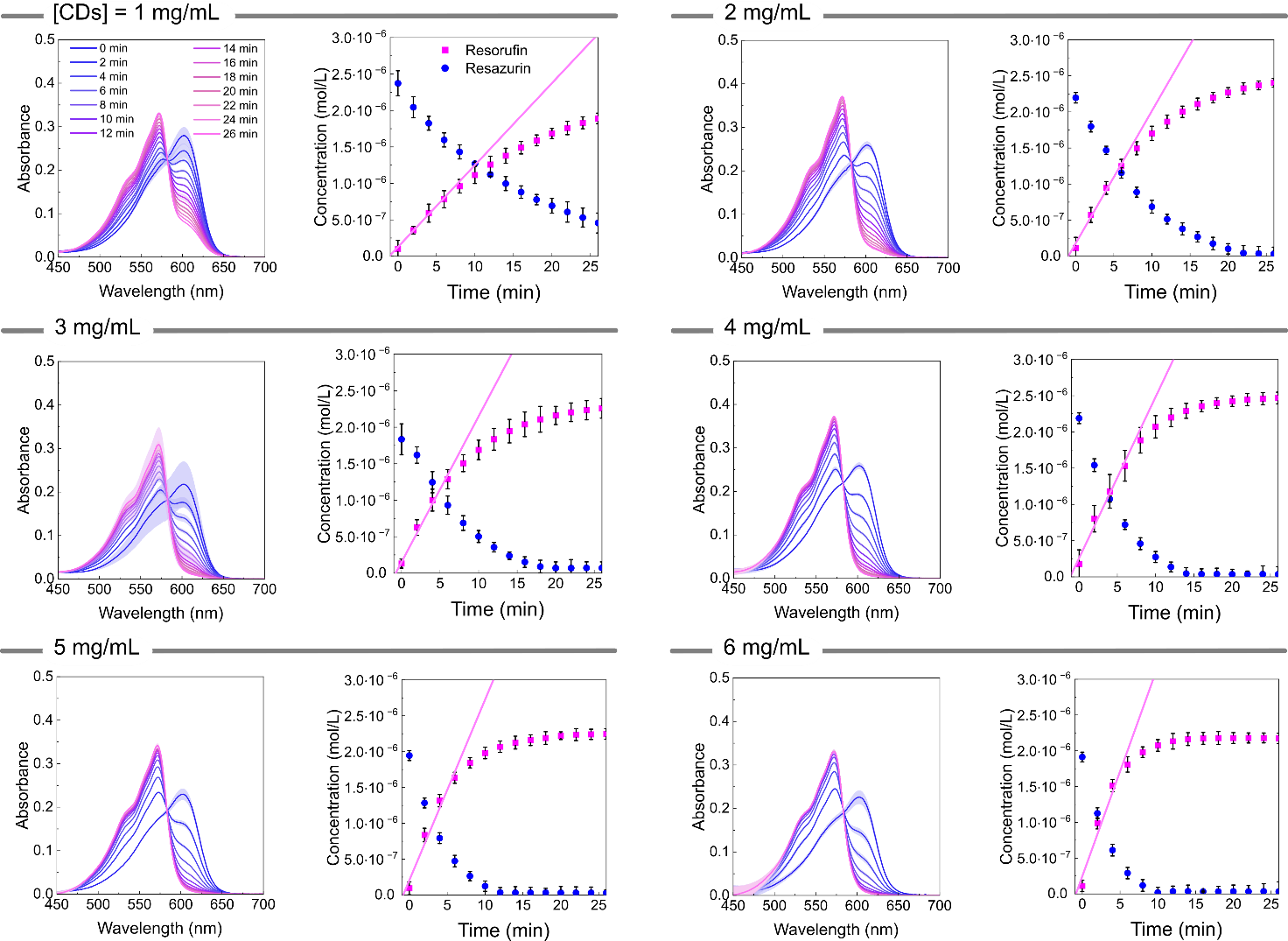


**Figure S11.** **CDs-1** mediated reduction of resazurin in the dark at pH 10.8. A solution containing **CDs-1** and resazurin in carbonate buffer (0.1 M, pH 10.8) was placed in a PMMA semi-micro cuvette (reaction volume = 1 mL), and the reaction was automatically monitored for 26 min by UV-Vis spectrophotometry, acquiring a spectrum every 2 min. The resazurin concentration was kept stable at 2x10⁻⁶ M, while the **CDs-1** concentration was varied as indicated in the figure. The graphs reported are the time-dependent UV-Vis spectra acquired by the instrument and the derived plots of resazurin and resorufin concentration. Derived data were obtained according to **Equation 1** and **Equation 2**. The light pink lines reported in the graph refer to the linear fitting of the obtained data. Error bars and error bands refer to the standard deviation over three replicas.

### CDs-1 mediated reduction of resazurin (under light irradiation, pH 9.4)


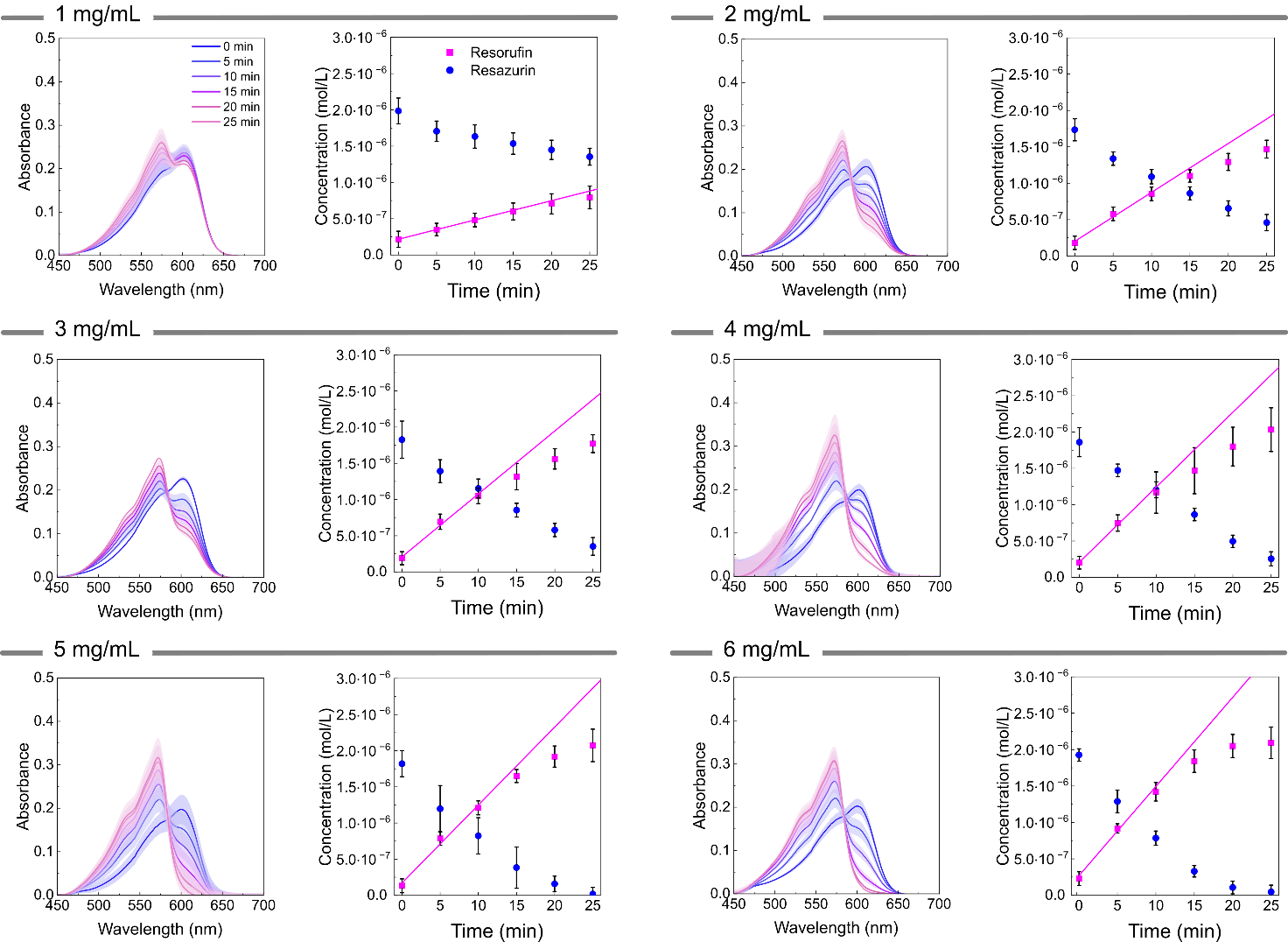


**Figure S12.** **CDs-1** mediated reduction of resazurin under light irradiation at pH 9.4. A solution containing **CDs-1** and resazurin in carbonate buffer (0.1 M, pH 9.4) was placed in a PMMA semi-micro cuvette (reaction volume = 1 mL), and the reaction was kept under light irradiation and monitored for 25 min by UV-Vis spectrophotometry by manually acquiring a spectrum every 5 min. A Kessil lamp (λ_max_ = 427 nm, 45 W, Irradiance = 200 mW/cm^2^) was used as visible light source at its maximum power, and in the absence of band filters. The resazurin concentration was kept stable at 2x10⁻⁶ M, while the **CDs-1** concentration was varied as indicated in the figure. The graphs reported are the UV-Vis time-dependent spectra acquired by the instrument and the derived plots of resazurin and resorufin concentration. Derived data were obtained according to **Equation 1** and **Equation 2**. The light pink lines reported in the graph refer to the linear fitting of the obtained data. Error bars and error bands refer to the standard deviation over three replicas.

## Quantum yield (QY) estimation

The QY of the resazurin reduction was estimated from the linear fitting of the data reported in **Table S3**.


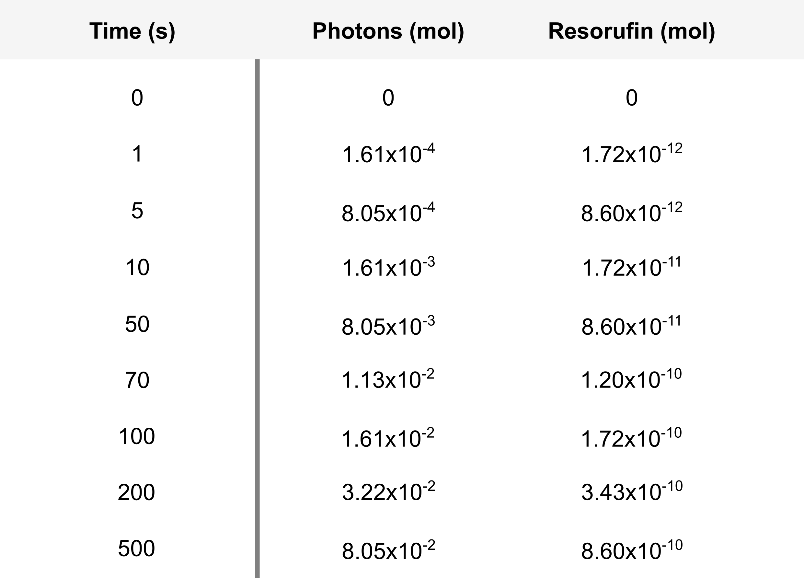


**Table S3**. Table reporting the moles of photons and the moles of resorufin (reaction product) at a specified kinetics timepoint (s). For QY estimation, the model reaction chosen was the following: **CDs-1** mediated resazurin reduction in carbonate buffer (0.1 M, pH 9.4) under light irradiation (Kessil lamp, λ_max_ = 427 nm, 45 W, Irradiance = 200 mW/cm^2^), where [**CDs-1**] = 4 mg/mL and [resazurin] = 2x10^-6^ M.

**Figure S13.** Graph showing the linear correlation between the moles of incoming photons (x axis) and the moles of obtained product (resorufin, y axis). The plotted data refers to the chosen model reaction of resazurin reduction: **CDs-1** mediated resazurin reduction in carbonate buffer (0.1 M, pH 9.4) under light irradiation (Kessil lamp, λ_max_ = 427 nm, 45 W, Irradiance = 200 mW/cm^2^), where [**CDs-1**] = 4 mg/mL and [resazurin] = 2x10^-6^ M. Blue line refers to the linear fitting of the data, where the angular coefficient corresponds to the QY. Blue band represents the 95% confidence interval of the data.

From the linear fitting of the data reported in **Table S3**, a quantum yield of 1.07x10^-8^ was found (**Figure S13**). Since this number was found to be smaller than 1, the reduction of resazurin was hypothesized to proceed without the involvement of radical chain mechanism.^3,4^

## CDs-1 reusability


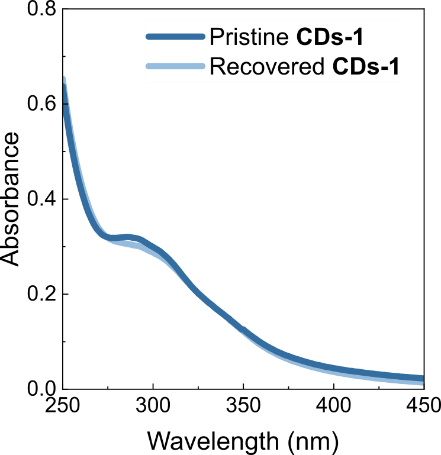


**Figure S14**. Comparison of UV-Vis spectra of **CDs-1** before and after the redox assessments. **CDs-1** after the reaction were recovered by dialyzing the reaction mixture for 48 h against pure Milli-Q water (MWCO = 500-1000 Da). After dialysis, the **CDs** were lyophilized and analyzed with UV-Vis spectroscopy. The UV-Vis spectrum of pristine **CDs-1** is reported for comparison.

## Zeta-potential measurements of CDs 1-3


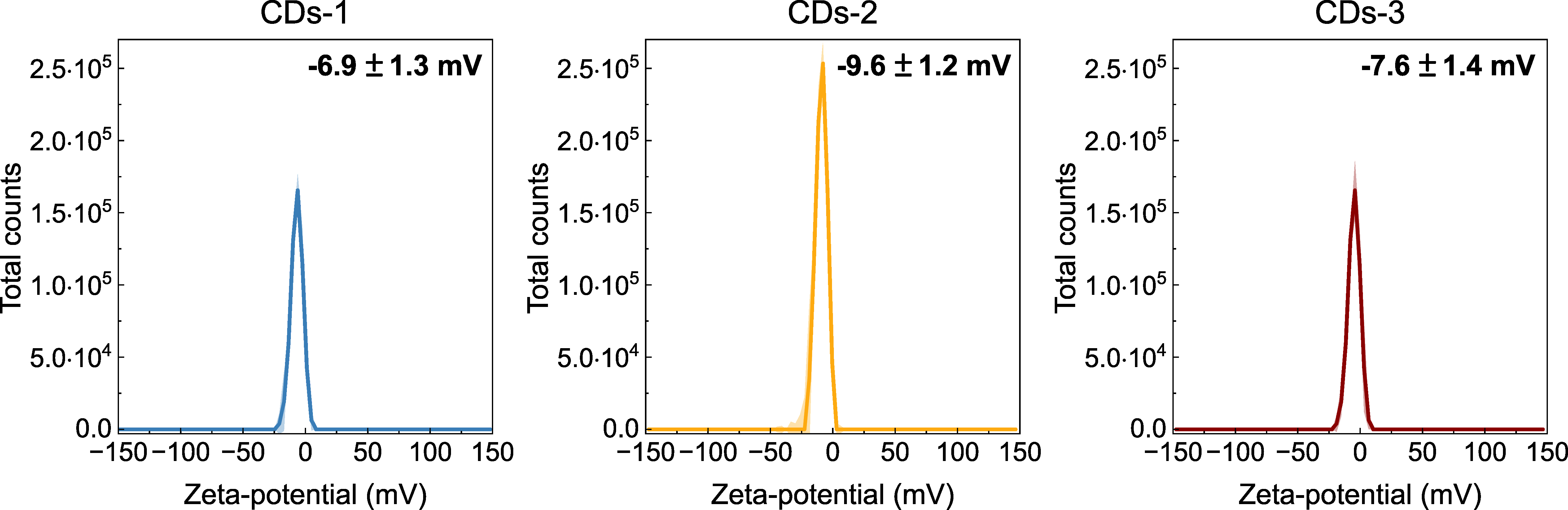


**Figure S15**. Plot comparing the Zeta-potential values for **CDs 1-3**. Samples were analyzed at the concentration of 4 mg/mL in carbonate buffer (0.005 M, pH 9.4). The average Zeta-potential of **CDs 1-3** is indicated in each plot. Errors bands refer to the standard deviation over three measurements of the same **CDs** batch.

## Cyclic voltammetry


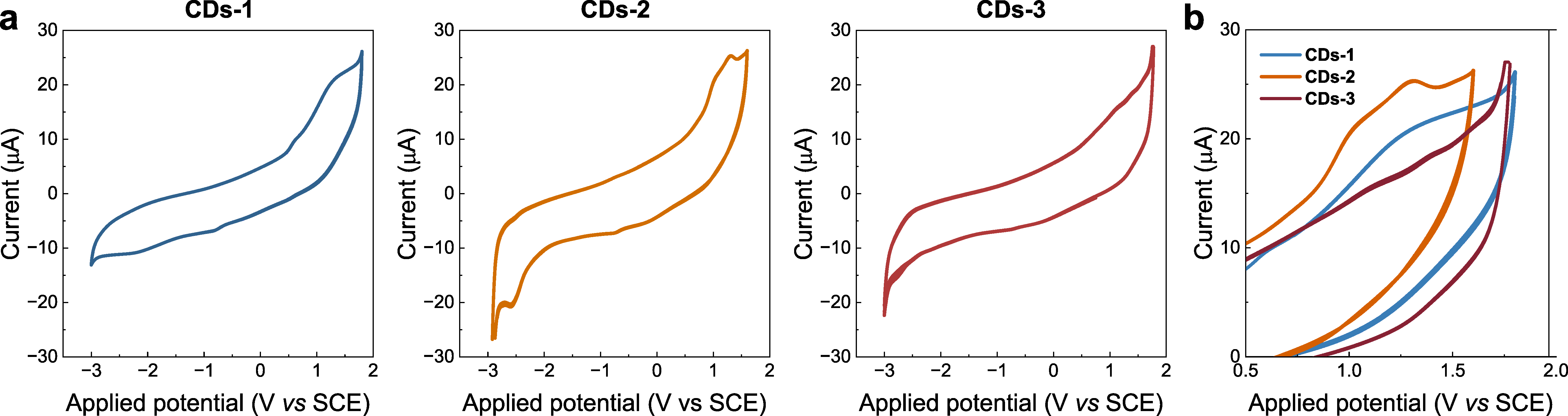


**Figure S16. a)** Cyclic voltammograms of **CDs 1-3** in DMF (0.1 M TBAPF₆). **b)** Magnified oxidation region. Working electrode: glassy carbon (3 mm diameter); counter electrode: platinum wire; reference electrode: saturated calomel electrode (SCE). Oxygen was removed by purging the DMF solution with Argon.


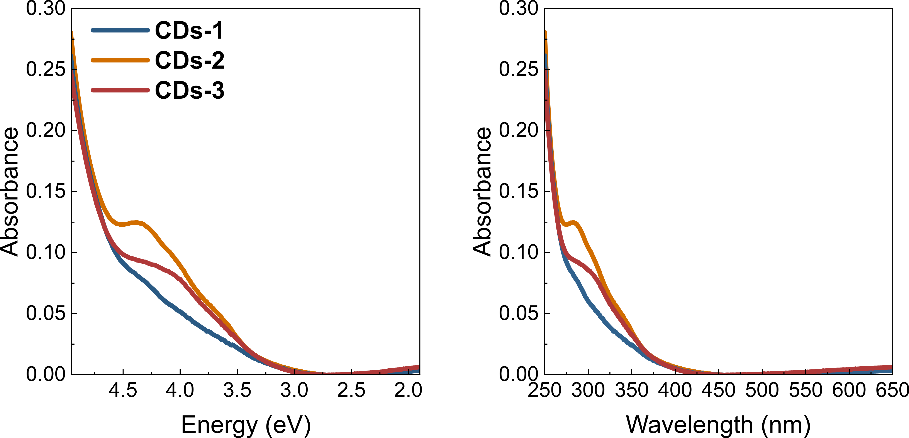


**Figure S17.** Absorption spectra of a solution 10^-3^ mg/mL of **CDs 1-3** in Milli-Q water reported as a function of energy (eV). Traditional absorption spectra of the same solutions are reported on the right graph for comparison.

## ****Control experiments with molecular models****

Control experiments were conducted to assess the reductive abilities of small molecules and polymers bearing amines and/or carboxylic acid moieties, demonstrating their ineffectiveness in reducing resazurin to resorufin. Results are reported in **Figure S18** and **Figure S19**. Of all the molecules and small polymers tested as potential reducing agents, PEI was the only one that showed a reducing effect on resazurin (**Figure S19**).


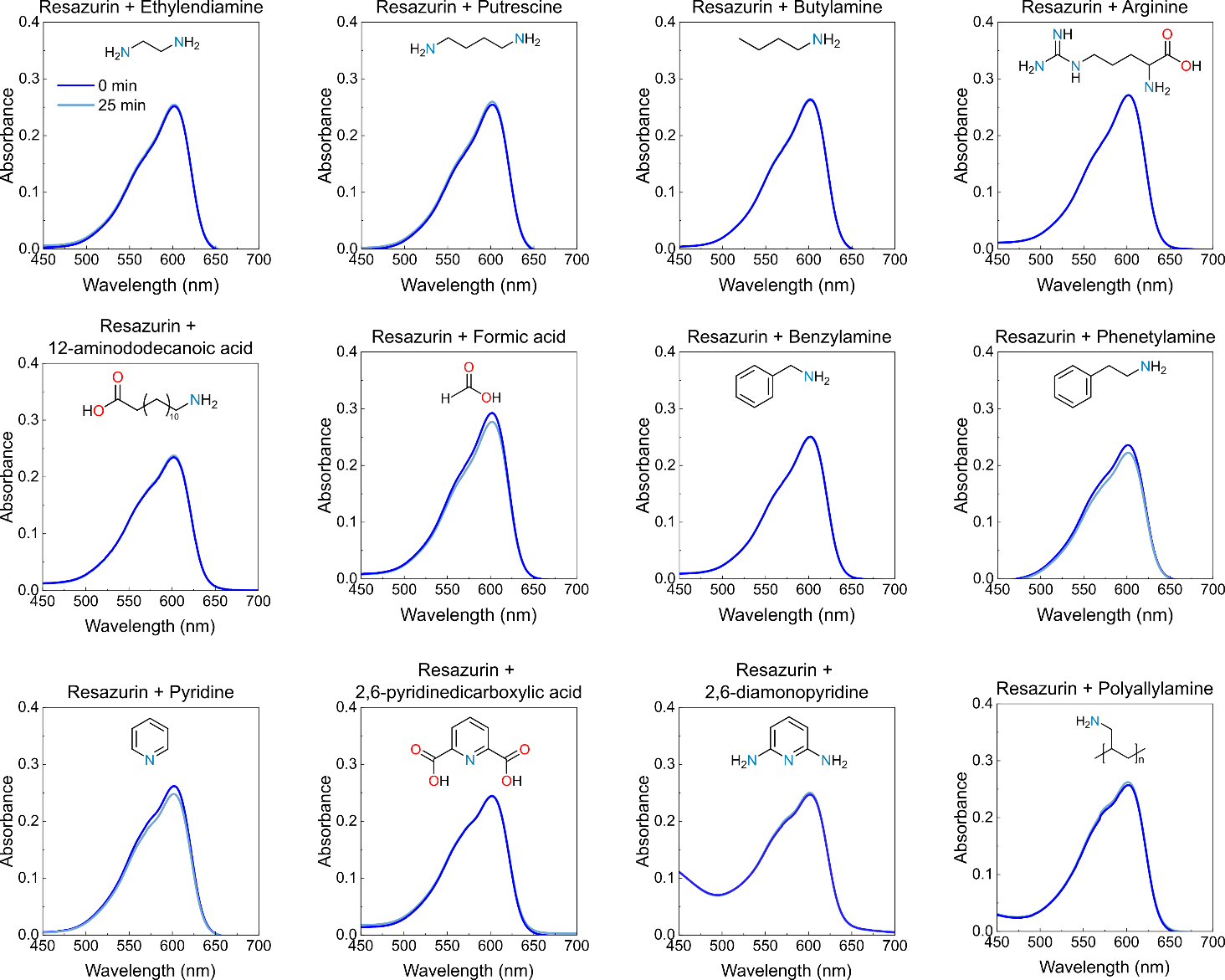


**Figure S18.** UV-Vis absorbance spectra of a solution of resazurin (2x10^-6^ M) in carbonate buffer (0.1 M, pH 9.4) acquired in the presence of different molecular systems. Each molecular system is reported on the top of the corresponding spectrum and was tested at the concentration of 5.4×10^-3^ M, matching the surface amine concentration of 4 mg of **CDs** in 1 mL of reaction mixture. All the tests were carried out inside a PMMA semi-micro cuvette (reaction volume 1 mL), and automatically monitored by acquiring a UV-Vis spectrum at t_0_ (dark blue spectrum) and after 25 minutes (light blue spectrum).


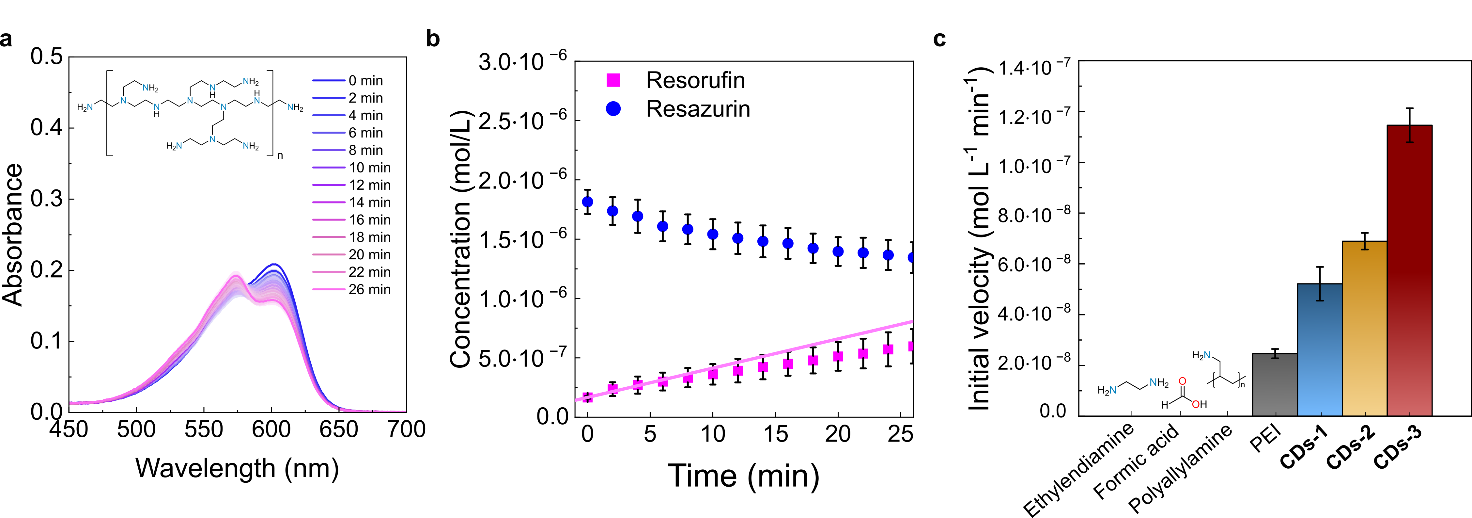


**Figure S19.** PEI-mediated reduction of resazurin in carbonate buffer (0.1 M, pH 9.4, resazurin concentration 2x10^-6^ M). The reaction was carried out inside a PMMA semi-micro cuvette (reaction volume 1 mL) and was automatically monitored for 26 min by UV-Vis spectrophotometry, acquiring a spectrum every 2 min. **a)** Plot showing the time-dependent UV-Vis spectra acquired by the instrument. **b)** Derived plots of resazurin and resorufin concentration obtained according to **Equation 1** and **Equation 2**. **c)** Plot comparing the initial velocities of resazurin reduction in the presence of different molecular systems (ethylenediamine, formic acid, polyallylamine, PEI, and **CDs 1-3**). Ethylenediamine and formic acid were used at the concentration of 5.4×10^-3^ M, matching the surface amine concentration of 4 mg of **CDs** in 1 mL of reaction mixture. Polyallylamine and PEI were tested at the concentration of 4 mg/mL, corresponding to an amine concentration inside the reaction mixture of 70.0x10^-3^ M and 11.6x10^-3^M respectively. Error bars and error bands refer to the standard deviation over three replicas.

## ^1^H-NMR binding experiments

No chemical shift changes were observed when ethylenediamine or formic acid replaced **CDs** in the resazurin solution (**Figure S20**).


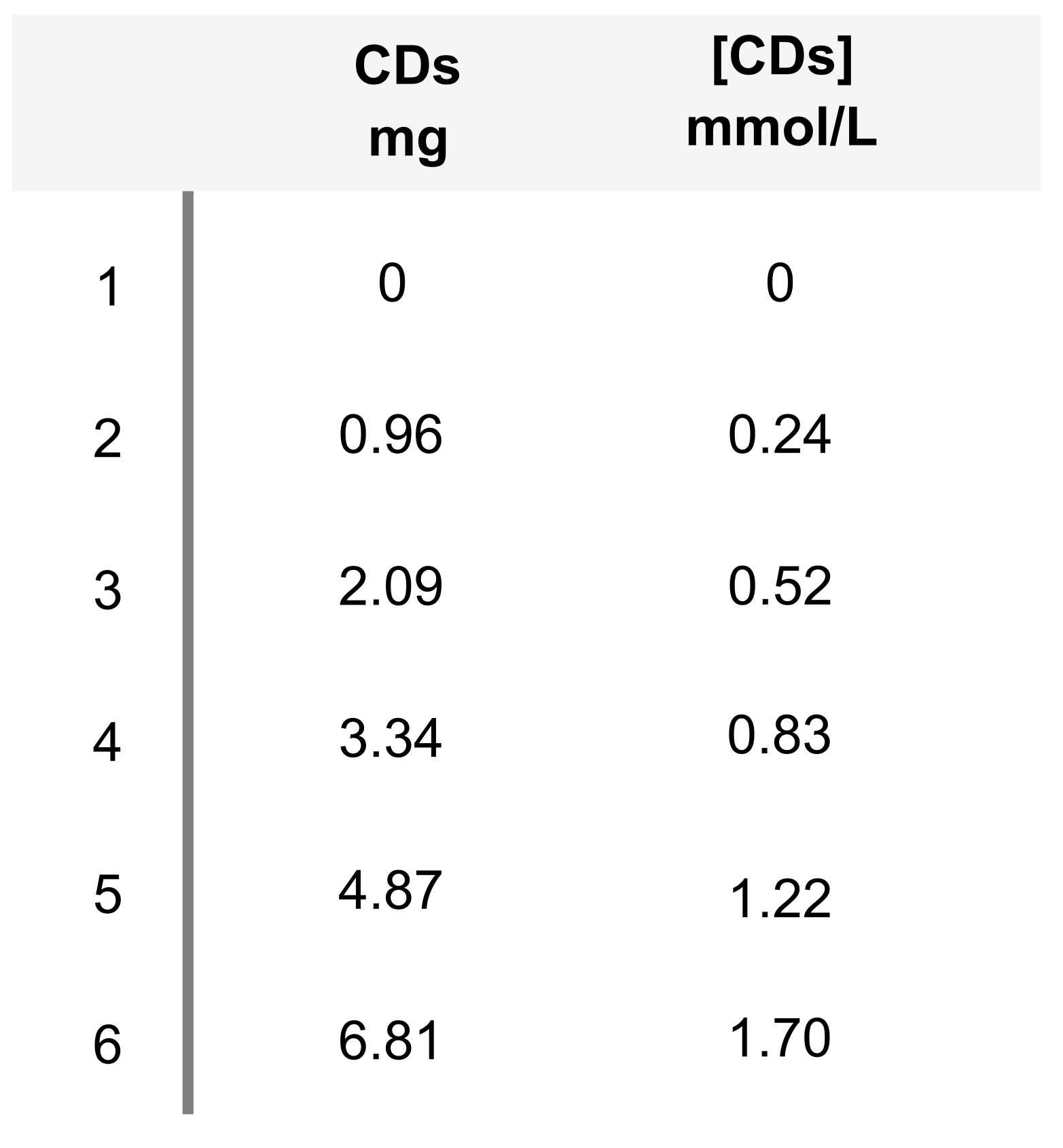


**Table S4.** Table showing the amount of **CDs-1** added to resazurin for each titration experiment. The second column indicates the molar concentration of the **CDs** in the final solution. The concentrations were calculated based on the molecular weight (M_n_) of **CDs-1** calculated by GPC.


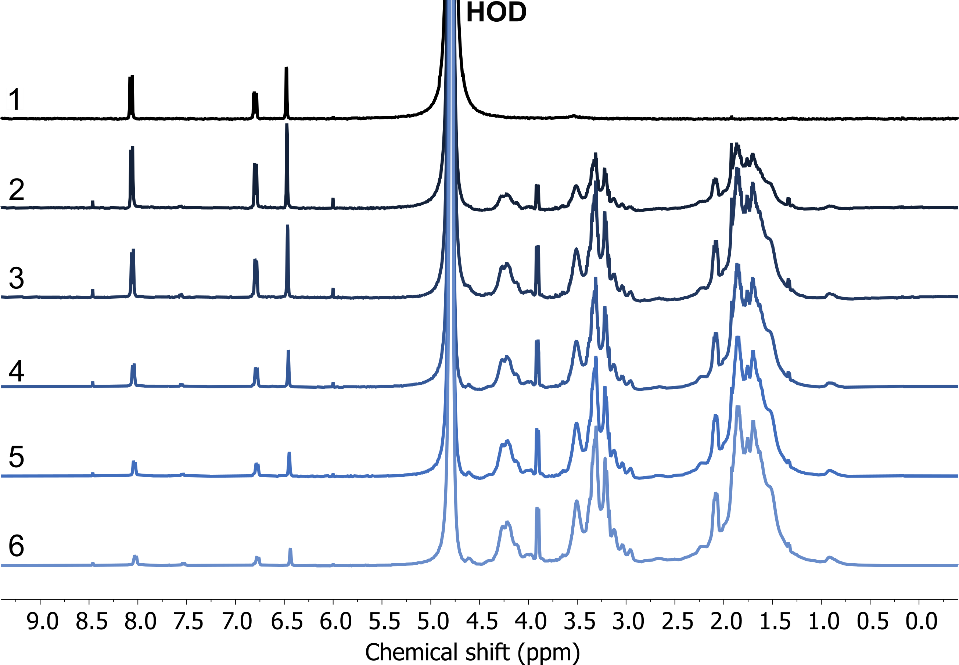


**Figure S20.** Comparison of ¹H-NMR spectra of pure resazurin and with increasing **CDs-1** concentrations. **CD** quantities for each spectrum are listed in **Table S4**. All spectra were acquired in D_2_O carbonate buffer (0.1 M, pH 9.4) and calibrated against residual solvent.

As can be seen in the ^1^H-NMR spectra reported in **Figure S20**, three small peaks ascribable to the formation of the resorufin product can be found at 8.46 ppm, 7.52 ppm, and 6.00 ppm. Fast resazurin reduction occurring upon **CDs-1** complexation prevented complete binding site saturation, precluding precise *K_D_* determination. The binding constant was therefore estimated from available experimental data (**Figure 4**). Moreover, resorufin may also display a partial affinity for **CDs-1**, therefore competing with resazurin for the saturation of **CD** binding sites.


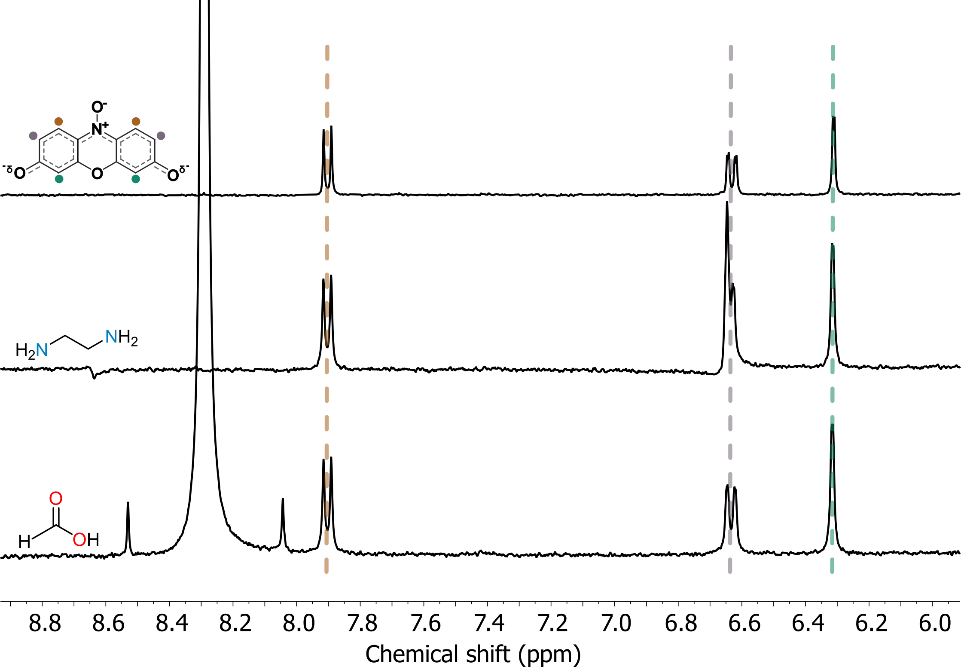


**Figure S21.** Comparison of ^1^H-NMR spectra of pure resazurin, ethylenediamine, and formic acid. No chemical shift changes in the resazurin signals were observed. The molar concentration of ethylenediamine and formic acid (5.4×10^-3^ M) matched the surface amine concentration of 7 mg **CDs-1** in 800 μL solution (maximum amount from the previous experiment). All spectra were acquired in D₂O carbonate buffer (0.1 M,pH 9.4) and calibrated against residual solvent.

## Isothermal titration calorimetry (ITC) measurements


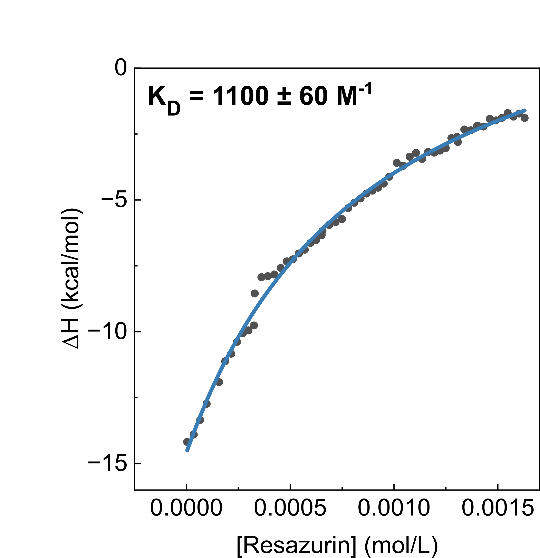


**Figure S22.** Plot showing the fitting of the ITC data ([Resazurin] vs enthalpy), and the associated binding constant. Experiments were performed in carbonate buffer (0.1 M, pH 9.4) with resazurin (2x10^-6^ M) and **CDs-1** (0.8x10^-3^ M). The error on K_D_ was calculated based on the error on K_d_ retrieved from the fitting.

ITC measurements require rigorous controls due to their sensitivity to any heat-producing processes that could compromise experimental precision. **Figure S23** presents control titrations of **CDs-1** *vs* buffer, buffer *vs* buffer, and resazurin *vs* buffer. These controls confirmed complete dissolution of all components (no endothermic dissociation peaks were detected) and demonstrated negligible heat intensities compared to the **CD**-resazurin titration experiment.


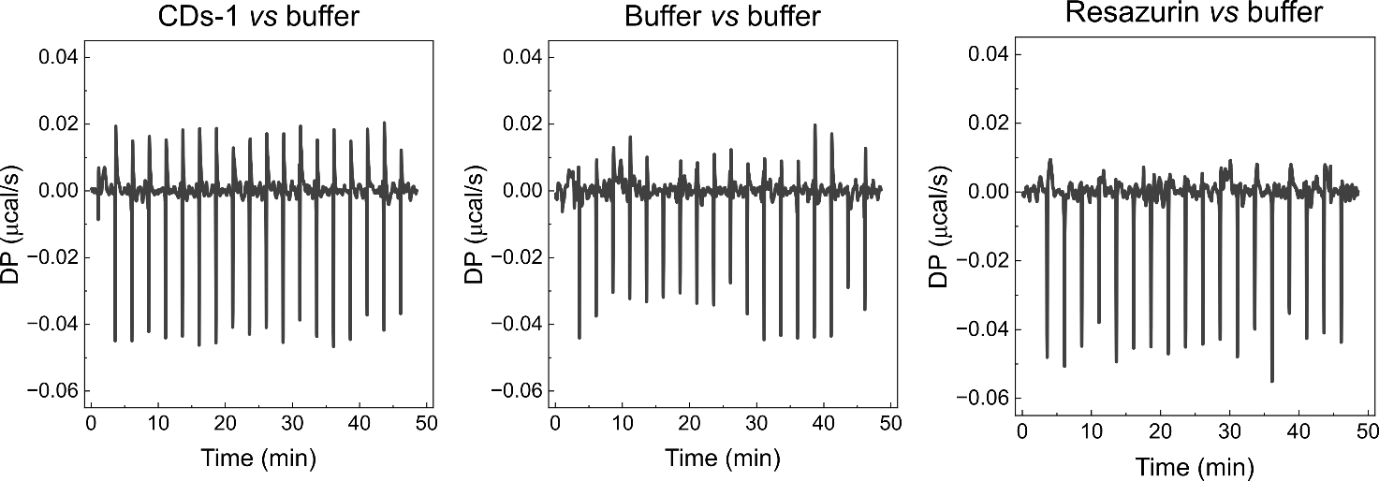


**Figure S23.** Blank titration controls showing ITC thermograms for **CDs** titrated with buffer, buffer titrated with buffer, and resazurin titrated with buffer. All three controls showed negligible heat changes compared to resazurin-**CD** titrations (**Figure 5a**). Experimental conditions: carbonate buffer (0.1 M, pH 9.4); resazurin 2x10^-6^ M; **CDs-1** 0.8x10^-3^M (4 mg/mL).

# Bibliography

1. Njoku, D. I.; Guo, Q.; Dai, W.; Chen, J. L.; Mao, G.; Sun, Q.; Sun, H.; Peng, Y.-K., The Multipurpose Application of Resazurin in Micro-Analytical Techniques: Trends From the Microbial, Catalysis and Single Molecule Detection Assays. *TrAC* **2023,** *167*, 117288.

2. Khazalpour, S.; Nematollahi, D., Electrochemical Study of Alamar Blue (Resazurin) in Aqueous Solutions and Room-Temperature Ionic Liquid 1-butyl-3-methylimidazolium tetrafluoroborate at a Glassy Carbon Electrode. *RSC Adv.* **2014,** *4* (17), 8431-8438.

3. Chan, A. Y.; Perry, I. B.; Bissonnette, N. B.; Buksh, B. F.; Edwards, G. A.; Frye, L. I.; Garry, O. L.; Lavagnino, M. N.; Li, B. X.; Liang, Y., Metallaphotoredox: the Merger of Photoredox and Transition Metal Catalysis. *Chem. Rev.* **2021,** *122* (2), 1485-1542.

4. Buzzetti, L.; Crisenza, G. E. M.; Melchiorre, P., Mechanistic Studies in Photocatalysis. *Angew. Chem. Int. Ed.* **2019,** *58* (12), 3730-3747.
